# A Group 6 LEA Protein Plays Key Roles in Tolerance to Water Deficit, and in Maintaining the Glassy State and Longevity of Seeds

**DOI:** 10.1111/pce.15649

**Published:** 2025-06-05

**Authors:** Inti A. Arroyo‐Mosso, H. Nicholay Diaz‐Ardila, Alejandro Garciarrubio, U. G. V. S. S. Kumara, David F. Rendón‐Luna, Teresa B. Nava‐Ramírez, Thomas C. Boothby, José Luis Reyes, Alejandra A. Covarrubias

**Affiliations:** ^1^ Departamento de Biología Molecular de Plantas, Instituto de Biotecnología Universidad Nacional Autónoma de México Cuernavaca Morelos Mexico; ^2^ Departamento de Ingeniería Celular y Biocatálisis, Instituto de Biotecnología Universidad Nacional Autónoma de México Cuernavaca Morelos México; ^3^ Department of Molecular Biology University of Wyoming Laramie Wyoming USA

**Keywords:** *Arabidopsis thaliana*, intrinsically disordered proteins, LEA proteins, seed longevity, water deficit

## Abstract

Plants have a wide range of adaptive and protective mechanisms to cope with dehydration. Central in these processes are the Late Embryogenesis Abundant (LEA) proteins, whose levels notably increase in response to dehydration during seed development and vegetative tissues. Understanding the function of LEA proteins is essential for gaining insights into plant development and their adjusting responses to environmental stress. This study focuses on Group 6 LEA proteins (LEA6) from *Arabidopsis thaliana*: AtLEA6‐2.1, AtLEA6‐2.2, and AtLEA6‐2.3. Phylogenetic analysis reveals that LEA6 family emerged with seed plants, pointing to a unique role in seed viability. Functional characterization using T‐DNA insertion mutants demonstrated that AtLEA6‐2.1, but not AtLEA6‐2.2, is essential for tolerance to high‐osmolarity and salinity during germination and post‐germination growth. AtLEA6‐2.1 deficiency also altered root architecture under salinity, increasing primary root length while reducing lateral root number and length, suggesting a role in root development not described before for a LEA protein. Furthermore, AtLEA6‐2.1 is critical for seed longevity, as mutants lacking this protein showed reduced germination after natural and accelerated aging. These mutants exhibited increased glass‐former fragility, indicating that AtLEA6‐2.1 deficiency reduces cellular viscosity, which we found correlates with reduced longevity. Our investigation extends to protective protein assays under dehydration, revealing that the acidic nature of this protein family requires specific conditions for its In Vitro protective activity. Overall, this study underscores the essential role of AtLEA6‐2.1 in the plant response to low‐water availability, seed longevity, and glassy state properties, making it a potential target for enhancing plant resilience to environmental challenges.

## Summary

- We show that a Group 6 LEA protein from *Arabidopsis thaliana* play key roles in plant responses to water deficit caused by environmental factors and developmental programs and help to preserve cellular integrity during desiccation maintaining seed aging. Accordingly, this LEA protein participates in preserving cellular viscosity in dry seeds.

## 1 Introduction

The production of seeds was one of the most successful events during plant evolution, allowing plant reproduction, plant dispersal, and persistence. The extreme climate changes that occurred during the Earth evolution led to the selection of structural and biological properties in seeds that enable them to endure harsh environmental conditions. Orthodox seed maturation involves a gradual and controlled loss of water, culminating in desiccation—a critical adaptation that allows seeds to remain viable for extended time periods. The ability of seeds to tolerate desiccation was a crucial adaptation, as long seed longevity—the duration for which a seed remains viable for germination—has been key for the dispersal of plant species across the Earth's surface and is essential for the conservation of plant genetic resources (Covarrubias et al. 2020; Linkies et al. 2010). Various physiological, molecular, and metabolic adaptations during the late stages of seed development are required for the plant embryo to survive in the dry state. These processes involve changes in the seed's structural composition and cellular metabolism. As most cells dry, they undergo a liquid‐to‐solid phase transition. Because of the heterogeneous composition of the cytosol, cells typically do not crystalize upon transition to the solid‐state, but rather form noncrystalline, or ‘glassy,’ solids (Angell 1995; Buitink and Leprince 2008). The ability of glasses to preserve protein structure and function and to prevent damage to other cellular components has been widely observed both In Vivo and In Vitro across various systems (Crowe et al. 1998; Kumara et al. 2024; Ramirez et al. 2024; Sakurai et al. 2008). It has also been proposed that the glassy state plays a vital role in seed longevity (Ballesteros and Walters 2019; Buitink and Leprince 2008).

Some of the events occurring during seed desiccation also take place in vegetative tissues under water deprivation (Bray 1997). These include changes in abscisic acid (ABA) levels, chromatin organization, and the accumulation of reserves, soluble sugars, heat shock proteins (HSPs), and LEA proteins, among other responses. The high abundance of LEA proteins during this developmental stage, and their presence in response to water deficit in vegetative tissues, led to the proposal for a role of these proteins in plant desiccation and/or dehydration tolerance (Bartels et al. 1988; Dure. 1993). Despite several efforts aimed at elucidating the In Vivo function of this set of proteins, their activities *in planta* remain elusive. Numerous In Vitro studies indicate that LEA proteins have protective roles against dehydration for cellular structures, proteins, and other macromolecules (Goyal et al. 2005; Hara et al. 2001; Popova et al. 2015; Reyes et al. 2005). The sensitivity to water limiting conditions of mutants in some *LEA* genes and the tolerance shown by their ectopic overexpression support their requirement for an optimal plant response to these stress conditions (Hernández‐Sánchez et al. 2022).

Although LEA proteins share common physicochemical properties, they can be classified into different families based on their amino acid sequences and the presence of conserved sequence motifs. This allows their classification in at least seven groups or families (Battaglia et al. 2008). Except for Group 5 LEA proteins, the other six groups show a biased amino acid composition, lack or have low abundance of hydrophobic residues and cysteines, and a high representation of hydrophilic and/or charged amino acids. These characteristics are consistent with their propensity to exhibit high structural disorder in aqueous solution, which has been demonstrated for LEA proteins from different groups (for review Hernández‐Sánchez et al. 2022).

In this study, we addressed the role of the LEA protein family 6 (LEA6) in *Arabidopsis thaliana*. This LEA protein family exhibits highly conserved motifs among different species of vascular plants and its members have relatively small molecular mass (7–14 kDa) (Battaglia et al. 2008). LEA6 proteins were first described in *Phaseolus vulgaris* (PvLEA18, recently renamed as PvLEA6) (Colmenero‐Flores et al. 1997; Colmenero‐Flores et al. 1999), where there is only one *LEA6* gene. In *P. vulgaris*, LEA6 protein accumulates in high levels in dry seeds and pollen, and in response to water deficit in roots and shoots during vegetative growth. Likewise, *PvLEA6* gene expression is induced by ABA treatments, consistent with the presence of ABA‐responsive*‐*elements in its promoter region. The *PvLEA6* 3′‐UTR has an enhancing effect on protein accumulation (Battaglia et al. 2008; Moreno‐Fonseca and Covarrubias 2001). PvLEA6 responds to changes in water availability during plant cell growth by increasing its transcript and protein levels in the elongation regions of hypocotyls and roots under well‐irrigated conditions, where elongating regions exhibit less negative water potentials compared to mature regions (Colmenero‐Flores et al. 1999). Immunolocalization experiments showed that upon stress, PvLEA6 localizes to the cytosol and nucleus of all tissues, accumulating more in the vascular system. Structurally, PvLEA6 is intrinsically disordered in aqueous solution but gains slight helicity under macromolecular crowding and high osmolarity. It can also form homodimers both In Vitro and In Vivo (Rivera‐Najera et al. 2014).

To explore the role of the LEA6 protein family in plant responses to water deficit, this study focuses on the functional characterization of this protein family in *A. thaliana*, a model organism for most plants. This protein family includes three members: AtLEA6‐2.1 (At2g23110), AtLEA6‐2.2 (At2g23120), and AtLEA6‐2.3 (At2g33690). Our analyses show that LEA6 proteins are highly conserved and widely distributed in seed vascular plants; however, LEA6‐related proteins were not found in seedless vascular or in nonvascular plants. Although transcripts of two *AtLEA6* family members, *AtLEA6‐2.1* and *AtLEA6‐2.2*, accumulate in seeds and in response to water deficit treatments, genetic analysis of mutants in these two genes showed that only the AtLEA6‐2.1 protein is necessary to tolerate water limitation during germination and for maintaining seed longevity. Consistently, *atlea6‐2.1* seeds exhibited a defective vitrified state. The vitrified state is an amorphous solid, similar to glass, that reduces molecular mobility in cells, thereby helping to preserve cellular integrity during desiccation (Leprince et al. 2017). Furthermore, it was demonstrated that AtLEA6‐2.1 protein provides protective effects during In Vitro dehydration, particularly under more alkaline pH conditions than those typically used. This highlights the relevance of protein net charge and the electrostatic interactions in the In Vitro protection mechanisms of LEA proteins.

## 2 Materials and Methods

### 2.1 Finding the Broadest Distribution of LEA6 Proteins

To explore the distribution of LEA6 proteins, we obtained 373 sequences from the Pfam entry PF10714 as of February 2023 (Mistry et al. 2021). All these sequences originated from angiosperms (NCBI Magnoliopsida, Taxonomy ID: 3398). Additionally, we compiled 11 gymnosperm sequences from the literature, though their exact sources could not be retraced. This collection included seven sequences labeled as *Pinus*, two as *Picea*, and one each as *Ginkgo* and *Pseudotsuga*.

From the Angiosperm sequences, we derived the following consensus sequence:

“EEKKGKLEGLPLESSPYVKYKDLEDYKRKGYGTEGHLEPKPGRG”, containing the conserved motif ‘SPY–Y–LEDYK—YGT’. From the Gymnosperm sequences, we derived this consensus sequence:

“DSKPKTEVLPTESSSPYTNYKDLDDYKMRGYGAEGHVDPLQNKP”, which contains the related motif ‘SPY–Y–LDDYK—YGA’.

Using these consensus sequences, we conducted separate homologue searches with BLASTp against the Phytozome 13 proteomes and NCBI's nr protein database. These searches increased the number of unique LEA6 sequences to 496, all restricted to Angiosperms. To address potential limitations due to the default hit limits of BLASTp, we repeated searches in the nr database, explicitly excluding angiosperm proteins. Likewise, we focused the search in Phytozome by excluding monocot and eudicot proteomes. We also searched for LEA6 proteins in specific genomes and proteomes of gymnosperms. These included the *Pinus taeda* (Zimin et al. 2017) and *Pseudotsuga menziesii* (Neale et al. 2017) genomes, and the proteomes of *Ginkgo biloba* (Gb_09268, Ginkgo DB) (Habib et al. 2022), *Welwitschia mirabilis* (Wan et al. 2021), *Cycas panzhihuaensis* (Liu et al. 2022), *Torreya grandi*s (Lou et al. 2023) and *Pinus tabuliformis* (Niu et al. 2022). These searches were performed with MMseqs. 2 (Steinegger and Söding 2017).

### 2.2 Phylogenetic Reconstruction

For analyzing LEA6 family evolution, we focused on Angiosperm proteins from RefSeq, a uniform collection derived from *The NCBI Eukaryotic Genome Annotation Pipeline*. This study was limited to one representative organism per species, excluding subspecies and cultivars. The data set includes 186 proteins from 132 species across 27 taxonomic orders. Given the distinct selective pressures on intrinsically disordered regions compared to ordered proteins, our analysis concentrated on the conserved segment of LEA6 proteins that is the least disordered. We substituted original RefSeq sequence names with concise, informative identifiers, starting with a four‐part numerical code representing order, family, genus, and species, followed by a condensed GenusSpecies name and, if applicable, a letter indicating a paralogue. The conversion from RefSeq names to our naming system and the FASTA sequence of this collection, including the *Ginkgo biloba* lea_6 protein, is available in Supporting Information S2: Table S1.

To build the phylogeny of the 186 LEA6 proteins from angiosperms, plus one from Ginko, we followed these steps: protein sequences were aligned with MAFFT (Katoh and Toh 2010), and the resulting alignment was first trimmed with ClipKIT (Steenwyk et al. 2020) using option ‐m gappy, and then by hand using Jalview (Waterhouse et al. 2009) to ensure that the angiosperm consensus described above remained uninterrupted. Then a phylogeny was built using RAxML‐NG (Kozlov et al. 2019) with 200 bootstrap replicates (command: raxml‐ng ‐‐all ‐‐msa alignment. fasta ‐‐model LG + G8 + F ‐‐tree pars(10) ‐‐bs‐trees 200). Using the same strategy, we also built order‐level phylogenies for 7 of the 27 taxonomic orders; those with at least 6 proteins and at least one species with 2 proteins (paralogues). These were Fabales, Poales, Brassicales, Malpighiales, Cucurbitales, Malvales and Myrtales. The global tree and the order‐level trees in Newick format with bootstrap values are shown in Supporting Information S2: Table S2.

### 2.3 Reconciliation of Protein and Species Trees

The standard strategy for deducing the history of duplication and losses in a protein family involves reconciling the protein tree with the species tree. To achieve this, we used the Treerecs software (Noutahi et al. 2016), which requires a fully resolved species tree. We obtained this from a list of TaxIDs from the NCBI taxonomy of the species, transforming it into a phylogeny with PhyloT (Letunic 2015) by arbitrarily resolving any polytomies. For this exercise, the Treerecs contraction threshold was set to 200. We also performed a reconciliation using SpeciesRax (Morel et al. 2022) from GeneRax, which can construct the species tree from the protein tree.

### 2.4 Plant Material and Growth Conditions

In this study, the *A. thaliana* Wassilewskija (Ws‐0) and Landsberg (Ler‐0) ecotypes were used. T‐DNA mutant lines for *AtLEA6‐2.1* in Ws‐0 background were obtained from the FLAG collection (FLAG584B07, FLAG579E12, and FLAG290C07, referred to here as FLAG_1, FLAG_2 and FLAG_3, respectively), while that for *AtLEA6‐2.2* (CSHL_ET9692) in Ler‐0 background was obtained from CSHL T‐DNA insertional mutant collection. All analyses were performed in homozygous lines, selected based on the corresponding resistance. In most experiments, we used seeds stored for no more than 1 month. Arabidopsis seeds were surface‐sterilized by successive washes starting with 100% ethanol (2 min), followed by a solution containing 40% Chloralex and 0.02% Triton X‐100 (10 min) (Sigma‐Aldrich) and finally MQ (Milli‐Q) water (six times). For seed production, plants were germinated in 0.5× MS (Murashige & Skoog, CAISSON) medium with MES at pH 5.7 with 1% sucrose and 0.65% agar (SIGMA). After 5 days, seedlings were transferred to pots (10 × 3 cm) containing a mixture of MIX 3 (Sunshine), Perlite (Termolite) and Vermiculite (VERMITE) (4:3:3), added with Osmocote Plus (15‐9‐12, ICL) (7.6 g/Kg substrate). Plants were irrigated with distilled water and grown in a growth room at 22°C with a 16 h/8 h (light/dark) photoperiod.

For germination experiments, seeds were incubated in the dark at 4°C for 3 days to break dormancy. Then, they were sown onto solid 0.5× MS medium with 1% sucrose and 0.65% agar (SIGMA) and transferred to a growth chamber at 22°C, approximately 65% relative humidity, and 16 h/8 h (light/dark) photoperiod. Germination was monitored by radicle emergence using a NIKON SMZ1500 stereomicroscope. For RT‐PCR analysis, we obtained total RNA from 7‐dag (days after germination) seedlings transferred to media containing NaCl (200 mM), mannitol (300 mM), and ABA (100 µM), which were collected after 48 h. For low humidity treatment, 7‐dag seedlings were transferred to a controlled humidity chamber (approx. 70% relative humidity) and collected after 24 h. For the phenotypic evaluation during post‐germination growth, 5‐dag, Arabidopsis seedlings were transferred to 0.5× MS medium with or without 75 mM NaCl. After 12 days of stress treatment, dry weight of complete seedlings was determined.

### 2.5 Root Architecture Analysis

For the analysis of root architecture, primary root length and lateral root number were scored. For this, seeds of all lines were germinated on solid MS medium; after radicle protrusion, seedlings were transferred to square 12.5 × 12.5 cm dishes containing 0.2× MS salts in MES pH 5.7 with 1% sucrose and 1% Bacto agar (Becton, Dickinson and Company), or in the same medium with NaCl 75 mM, and incubated in vertical position at 22°C, approximately 65% relative humidity, and 16 h/8 h (light/dark) photoperiod. For each experiment, 30 seeds per line and treatment were sown. Root length was measured by marking the root tip position of every plant every day at the same hour until the 11th day after transplant (dat). At this point, plates were scanned (EPSON Perfection V600 Photo scanner) and primary and lateral roots were measured using ImageJ (1.54 g). Analysis was performed with GraphPad Prism (version 10.3.0). These experiments were repeated at least three times.

### 2.6 Nucleic Acids Purification and PCR Assays

Genomic DNA was obtained by the CTAB (hexadecyltrimethylammonium bromide) method (Murray and Thompson 1980). RNA was removed with 1% RNase A (Sigma‐Aldrich) for 30 min at room temperature, followed by phenol‐chloroform (1:1) extractions (Thermo‐Fisher Scientific, 2023). Samples were resuspended in TE buffer (10 mM Tris pH 8, 1 mM EDTA) and quantified. Total RNA from seedlings was obtained using the Quick‐ RNA MiniPrep kit (Zymo Research), following the manufacturer's instructions. We extracted high‐quality RNA from seeds using the CTAB method (Acosta‐Maspons et al. 2019) adapted for Arabidopsis. Nucleic acids were quantified at 260 nm using a NanoDrop spectrophotometer (ND‐1000 V3.5.2). cDNA was synthesized from 1 µg of DNase‐treated RNA using RevertAid Reverse Transcriptase (Thermo‐Fisher Scientific) following the manufacturer's protocol. The PCR assays were performed according to standard conditions (20 µL) using 10–50 ng of DNA or 0.2 μL of the cDNA synthesis mix as templates. Specific oligonucleotides were used at 10 pmol/μL. We used eIF4 (AT3G13920) as a housekeeping control gene, with the corresponding specific primers (Supporting Information S2: Table S3). The reactions were performed using an Eppendorf thermocycler (MasterCycler Pro Vapor Protect). DNA fragments were analyzed by 1% agarose gel electrophoresis in TAE buffer (Tris‐Acetate 40 mM‐EDTA 1 mM pH 8.0).

### 2.7 Construction of Plasmids

All transgenic lines used in this study were obtained through Arabidopsis transformation using the pCambia 1304 vector. The DNA fragment containing *AtLEA6.2‐1* coding region (279 bp) was obtained by PCR from genomic DNA, using specific oligonucleotides (Supporting Information S2: Table S3) that contained *Nco*I and *Bst*EII restriction sites for subsequent ligation into pCambia vector that was linearized with the same enzymes. All constructs were verified by PCR analysis and DNA sequencing.

### 2.8 Plant Transformation and Selection

*A. thaliana* (Ws‐0) transgenic lines were obtained by floral‐dipping *Agrobacterium tumefaciens‐*mediated transformation (Clough and Bent 1998). The *pCambia/AtLEA6‐2.1* construct was transformed into the *A. tumefaciens* strain GV3010 and selected for kanamycin resistance. Positive colonies were analyzed by PCR using specific primers (Supporting Information S2: Table S3). *A. tumefaciens* cultures carrying the construct of interest were obtained from an isolated and verified colony to transform wild‐type or mutant Arabidopsis plants (FLAG_1). Transformants were selected by herbicide‐resistance using hygromycin B (25 µg/mL) and choosing seedlings with long hypocotyls (0.1 to 1.0 cm) after a growth regime of 4–6 h light/36 h darkness (Harrison et al. 2006). Seedlings were maintained in 16 h/8 h (light/dark) photoperiod for 24 to 48 h before being transferred to the substrate, where they were grown as described before. In all cases, we used T3 seeds to obtain homozygous lines for further analysis. The transgenic lines derived from the FLAG_1 mutant background (35S:*AtLEA6‐2.1*::*atlea6‐2.1*) were renamed as Comp_X, where X is a number assigned to refer to an independent transformation event. In the case of the transgenic lines derived from the wild‐type background (35S:*AtLEA6‐2.1*::Wt), they were designated as OverExpression lines (OE_X).

### 2.9 Accelerated Aging Treatment

The seed accelerated aging treatment was conducted as described by Bueso et al. (2014) using 1 month stored dry seeds (room temperature 20°C–25°C, 60% relative humidity). In this study, the heat treatment lasted for 12 h. After treatment, the seeds were sown on solid MS medium (0.5×) and germinated as described above. Germination capacity was determined by monitoring root emergence after 240 h.

### 2.10 Bacterial Protein Expression and Purification

For expression in *Escherichia coli*, we introduced the *AtLEA6.2‐1* ORF into the cloning vector pTYB11, using *Pst*I and *Sap*I restriction sites. The construction was confirmed by PCR analysis and sequencing. This vector, as part of the IMPACT‐ System (New England Biolabs Inc.), allowed us to overexpress and purify the AtLEA6.2‐1 protein without an affinity tag. The AtLEA6.2‐1 protein was expressed as a fusion with intein, and the chitin‐binding domain (CBD) in its N‐terminus. Subsequently, the recombinant protein was purified by affinity chromatography on a chitin column, with thiol treatment releasing AtLEA6.2‐1 protein from the intein and the chitin‐bound tag. PvLEA6 protein was obtained following the same protocol (Rivera‐Najera et al. 2014). After purification, the proteins were dialyzed against a suitable buffer, as needed, and kept frozen at −70°C or lyophilized for long storage. Proteins were quantified by the Qubit protocol using Qubit 4 Fluorometer (Thermo‐Fisher Scientific). The identity of all proteins used was verified by protein sequencing at Laboratorio Universitario de Proteómica‐UNAM.

### 2.11 In Vitro Partial Dehydration Assays

In Vitro partial dehydration assays were performed as described Rendon‐Luna et al. (2020). To determine if a protein can protect lactate dehydrogenase (LDH) from dehydration, LDH from rabbit muscle (Roche) was diluted to 0.25 μM (monomer) final concentration in 25 mM Tris‐HCl buffer at pH 7.5, with the protein to be tested, or without it, as a control. In some experiments, the pH of this mixture was modified as indicated. Importantly, the LDH activity was always performed at pH 7.5. In these assays, we used purified recombinant LEA proteins. The proteins to be tested were used at 10 times the molar concentration of LDH monomer. The mixture, in a final volume of 50 μL, was dehydrated in a SpeedVac concentrator (80 kPa, 25°C) (Savant SC200, Savant Instruments Inc.), and water loss was monitored by weight on a precision weighing scale. Dehydrated samples were rehydrated to the initial weight with Milli‐Q water. LDH activity was measured as described previously (Rendon‐Luna et al. 2020), adjusting the activity buffer to pH 7.5. Data from these assays were obtained from at least three independent experiments, using proteins from independent purification processes.

### 2.12 Measurement of Glass Transition Temperature (Tg)

Seed samples (less than 6 months of storage) were analyzed on a TA DSC 2500 instrument, using TRIOS software (TA Instruments TRIOS version #5.0.0.44608). Approximately 3.5 to 4.5 mg of seeds were placed in pre‐weighed differential scanning calorimeter (DSC) aluminum hermetic pans (TA 900793.901), hermetically sealed with aluminum hermetic lids (TA901684.901), and loaded in the DSC machine. Samples were heated by raising the temperature from 20°C to 250°C at a rate of 2°C/min. Using the Trios software (TA Instruments TRIOS version #5.0.0.44608), the Tg onset, midpoint, and endset temperatures for each seed line were obtained by identifying the point at which a change in the slope of the curve occurs.

### 2.13 Measurement and Calculation of Glass‐Former Fragility (m‐Index)

Glass‐former fragility was developed to assess how a system's viscosity changes as the system's temperature approaches the Tg (Angell 2002; Kumara et al. 2024), but it has recently been applied to measure how viscosity increases as water is lost (Crowley and Zografi 2001; Kumara et al. 2024; Ramirez et al. 2024). A system with a high glass‐former fragility does not gradually increase in viscosity as it dries; rather, it rapidly becomes viscous and vitrifies near the point of desiccation/vitrification. Conversely, a system with a low glass‐former fragility shows a gradual increase in viscosity as it dries (Angell 1995; Ballesteros and Walters 2019; Ramirez et al. 2024). Systems with a lower glass‐former fragility are generally considered more protective because they reduce molecular mobility, as the super‐viscous state, and the stabilization it provides, is induced earlier in the drying process. Hence, to determine the glass‐former fragility (m‐index) for each seed line, the glass transition temperature (Tg) was measured. Tg is the temperature at which a glassy or vitrified material regains some molecular mobility, exiting the immobile glassy‐solid state and entering a more rubbery‐solid state (Angell 1995; Grasmeijer et al. 2013; Ito et al. 1999). Generally, a higher Tg is associated with greater stability and capacity for protection (Lim and Hoag 2013; Mascia et al. 2020). The Tg onset marks the temperature at which molecular mobility begins to increase, providing insights into the initial relaxation dynamics of the system. In contrast, the Tg endset indicates the temperature at which the transition concludes, reflecting the thermal stability of the material. The m‐indices were calculated based on Equations 10 and 14 proposed by Crowley and Zografi (Crowley and Zografi 2001). In Crowley and Zografi's Equation 10, ‘m’ is the alternative fragility parameter, ‘ΔETg’ is the activation enthalpy of structural relaxation at Tg, ‘R’ is the gas constant, and ‘Tg’ is the experimental Tg onset temperature. In Equation 14, ‘ΔE*η*’ is the activation enthalpy for viscosity, ‘R’ is the gas constant, ‘Tg’ is the experimental Tg onset temperature, ‘Tgoff’ is the experimental Tg endset temperature, and constant (*k*) is an empirical constant of 5 (Crowley and Zografi 2001). An m‐index was calculated for each of the three replicates for each seed line, and these values were averaged to obtain mean m‐index for a given seed line.

(10) $$m=\frac{\left(\mathrm{\Delta ETg}\right)}{\left(\ln\;10\right)\mathrm{RTg}}$$

(14) $$k=\frac{\left(∆E\eta \right)}{\left(\frac{R\left(\frac{1}{\mathrm{Tg}-1}\right)}{\mathrm{Tgoff}}\right)}$$

### 2.14 Measurement of Water Content

Seed samples (approximately 3.5 to 4.5 mg) were loaded and run on a TGA5500 (TA Instruments) in pre‐tared platinum crucibles (TA 952018.906). The samples were heated from 30°C to 250°C at a rate of 2°C/min. The moisture level and the volatile contents were observed by monitoring the weight changes in TGA thermograms as temperature increases. Water loss from each seed sample, representing seven different lines, was calculated to determine the remaining water content. Mass differences were calculated considering the temperatures after ∼50°C but before the thermal denaturation at ∼150°C. The ‘Smart Analysis’ tool in TRIOS software (version 5.0.0.44608) was used to identify the inflection points between initial mass loss events and the plateau phase. Average values were calculated from triplicate measurements for each seed line.

### 2.15 Modeling Analysis at Different pH

The structural models of AtLEA6‐2.1 and LDH proteins were generated using the AlphaFold software, selecting the best ranked models (Jumper et al. 2021). These models were used to predict changes in the amino acid residue charges under different pH conditions. The patcHwork server (Schmitz et al. 2022) was used to identify amino acid residues with charge changes between pH 7 and pH 8. Docking simulations were performed using the HDock server (Yan et al. 2020) to explore potential modifications in the interactions between AtLEA6‐2.1 and LDHb due to the pH‐induced charge changes. The APBS (Adaptive Poisson‐Boltzmann Solver) server was employed to calculate the free energy and net charge of both proteins across different pH levels. This tool calculates the continuous electrostatic potential for large biomolecular systems (Jurrus et al. 2018).

### 2.16 Statistical and Computational Analyses

All the germination experiments included three to six replicates, each consisting of 30 to 100 seeds per replicate, using homozygous seeds from different batches. The data were fitted to sigmoidal dose–response curves. Arithmetic mean comparisons between two groups were analyzed using parametric Student's *t*‐tests, while arithmetic mean comparisons among multiple groups were performed using One‐Way ANOVA. The Tukey post hoc test was used to assess the significance of differences between pairs of individual group means. A *p‐*value greater than 0.05 was considered statistically not significant. All statistical analyses for the phenotypic assays were conducted using GraphPad Prism (version 10.3.0). The same statistical analysis was applied to the phenotypic data for seedling fresh and dry weights and for the assessed root parameters. The statistical data for the enzymatic protection assays were processed as reported by Rendon‐Luna et al. (2020). For the m Index and Tg analysis, one‐way ANOVA was used to compare measured and calculated values from different seed lines. The Tukey post hoc test was used to assess the significance of differences between pairs of individual group means. R (R 4.2.2) was used to calculate the one‐way ANOVA, Tukey's post hoc test, and *p*‐value results. In these experiments, all error values in bar graphs represent one standard error (SE). GraphPad Prism (version 10.3.0) was used to calculate the Pearson correlation and generate the corresponding graphs and error values representing the 95% confidence interval (CI) levels. The search and analysis of conserved sequence motifs were performed using the MEME Suite tools (v.5.5.7), while data representation was carried out with the ggmsa package (v.1.12.0) (Zhou et al. 2022) in R. The net charge per residue (NCPR), the fraction of charged residues (FCR), the isoelectric point, the molecular weight, and the hydropathy of the proteins were calculated using the CIDER algorithm (Holehouse et al. 2017), whereas the structural disorder was calculated using the IUPred2A algorithm (Erdős and Dosztányi 2020). We used the PLACE database (Higo et al. 1999) to search for *cis*‐elements located in the promoter regions of *AtLEA6‐2.1, AtLEA6‐2.2*, and *AtLEA6‐2.3* genes. The predicted *cis*‐elements were plotted using ggplot package. A description of the known or potential function of each *cis*‐element is provided in the Supporting Information S2: Table S4.

## 3 Results

### 3.1 LEA6 Protein Family Is Exclusive to Seed Plants

To examine the distribution of LEA6 proteins across the plant kingdom, we conducted a thorough search for the members of this family. At the start of this study, the PF10714 PFAM entry, corresponding to the LEA6 family, included 389 proteins. These involve two each from the basal angiosperms *Nymphaea colorata* and *Amborella trichopoda*. All the rest belong to the core angiosperms, with presence in 30 orders, 53 families, and 123 genera. As a LEA6 protein from *Pinus tabuliformis* has been reported (Zhou et al. 2022) we strived to find LEA6 family members outside the angiosperms. By searching in NCBI's nr database and in Phytozome 13, we found an additional 107 members of the family but still restricted to angiosperms. However, by searching specific databases and individual genome projects, we were able to find five *bona fide* LEA6 proteins from gymnosperms, including one each from *Ginkgo biloba*, *Welwitschia mirabilis*, *Pseudotsuga menziesii*, *Pinus taeda*, and *Pinus tabuliformis*, confirming the previous report. On the other hand, we did not find homologues in the proteomes of *Cycas panzhihuaensis*, *Torreya grandis* and *Thuja plicata* ‐the only Gymnosperm proteome included in Phytozome. Our results show that LEA6 proteins are widespread in angiosperms and have a presence in at least three of the four main sub‐classes of gymnosperms, although more sequenced genomes are needed to assess their prevalence in the later clade. Notably, LEA6 members were not found outside the seed plants, even though Phytozome includes the proteomes of four *Bryophyta*, nine *Chlorophyta*, one each from *Marchantiophyta*, *Polypodiophyta*, and *Rhodophyta*, and two species from the *Lycopodiopsida* class. Currently, LEA6 proteins appear to be restricted to seed plants, consistent with their possible function in safeguarding seed structures during desiccation.

### 3.2 Evolutionary Dynamics of the *LEA6* Gene Family in Angiosperms

**Figure 1:**
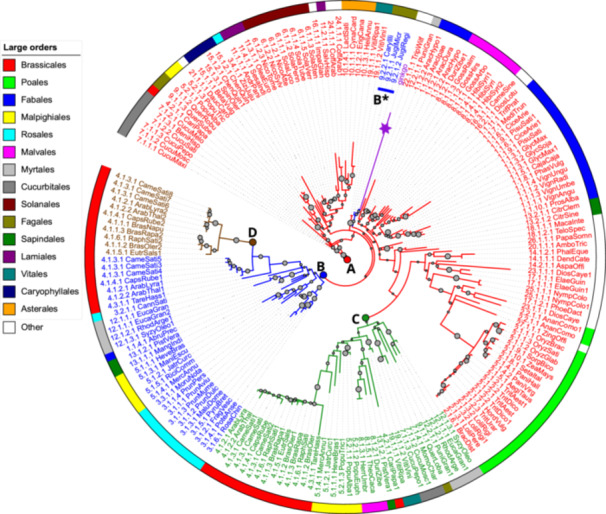
Phylogeny of the LEA6 family explained by ancestral duplications. Proteins are colored according to the proposed ancestral duplication from which they derive: A (the original LEA6) in red, B in blue, C in green, and D in brown. The region labeled ‘B*’ shows three proteins from the Ericales order that originate from duplication B but are probably misplaced due to their association with the long branch of *Ginkgo biloba* (indicated with a purple star). The size of the grey dots on the internal nodes indicates branch support. Colors in the external circle indicate the taxonomic order to which the proteins belong, as shown in the left key color's legend. Supporting Information S1: Figure S1 shows paralogous proteins per species.

We performed a phylogenetic reconstruction of a filtered collection of LEA6 protein sequences in angiosperms, including 186 proteins from 132 species, spanning 27 taxonomic orders (Figure 1 and Supporting Information S2: Table S1 and Table S5). We included the *G. biloba* LEA6 hoping its divergence would make it a suitable outgroup. However, RAxML placed it within the tree, as an ortholog of the LEA6 copy in orders that did not undergo duplications, like most monocots. If we force the tree to re‐root in *G. biloba*, the resulting tree becomes unnatural and more complex. Recognizing that intrinsically disordered regions face different selection pressures compared to ordered proteins, our investigation focused on the conserved region of LEA6 proteins, predicted to be the least disordered. A limitation of this approach is that this region is too short to resolve the history of 186 proteins unambiguously. Despite meticulous phylogenetic analysis, support for deep branches was weak. To address this problem, we constructed several protein trees focused on individual orders (Supporting Information S2: Table S2). As anticipated, these trees exhibited significantly higher average branch support, and their topology closely aligns with our best global tree. In addition to being consistent with the order‐level trees, the global tree shown in Figure 1 also parallels other trees we generated using different methods. The paralogues for each species are shown in Supporting Information S1: Figure S1.

To reconstruct the history of gene duplications and losses within the LEA6 family, we attempted to reconcile the protein tree with the species tree using two different software packages. Both attempts resulted in overly complex histories difficult to analyze. Treerecs proposed 45 duplications and 123 losses (Supporting Information S1: Figure S2), while SpeciesRax identified 18 duplications, 19 post‐speciation losses, and 19 horizontal gene transfer events (Supporting Information S1: Figure S3). By focusing on duplications that impacted multiple current species, we propose that all extant proteins descend from either the founding angiosperm LEA6 protein or from three key ancestral duplications: at the root of the angiosperms tree, there was a single copy A (Figure 1). Two early duplications created copies B and C. The copy C duplication likely occurred first, affecting nine orders: *Brassicales*, *Malpighiales*, *Cucurbitales*, *Malvales*, *Fagales*, *Myrtales*, *Rosales*, *Sapindales*, and *Vitales*. Subsequently, copy B was created, affecting a subset of those orders: *Rosales*, *Brassicales*, *Malpighiales*, *Myrtales*, and *Sapindales* (Figure 1). This scenario requires that copy C was lost from *Rosales* at some point. A final duplication within the *Brassicales* order created copy D (Figure 1), impacting species in the *Brassicaceae* family (4.1.x.y) (Supporting Information S1: Figure S4). The ancient duplications affected only 9 of 27 orders, while the other 18 show descendants of the ancestral copy A (Figure 1). This is particularly true for all monocot orders in the study (*Arecales*, *Asparagales*, *Dioscoreales*, *Poales*, *Zingiberales*), though recent duplications have created paralogs in some monocot species (Figure 1 and Supporting Information S1: Figure S1).

These events, along with more recent gene duplications and losses, explain the overall tree topology. Recent duplications appear in many branches (Figure 1). For example, a recent ancestor of *Camelina sativa* (4.1.3.1_CameSati from the *Brassicales* order) had three paralogs, each undergoing a triplication, resulting in nine paralogs. Gene losses are also common (Figure 1, Supporting Information S1: Figure S4). While the three ancestral duplications suggest *Brassicales* should have four paralogs, no single species in this order retains the complete set, with most having three or two of the four copies (Supporting Information S1: Figure S4). Altogether, these results show the great dynamism of this protein family, both at the short and long evolutionary times.

### 3.3 Physicochemical Properties of LEA6 Protein Family

**Figure 2:**
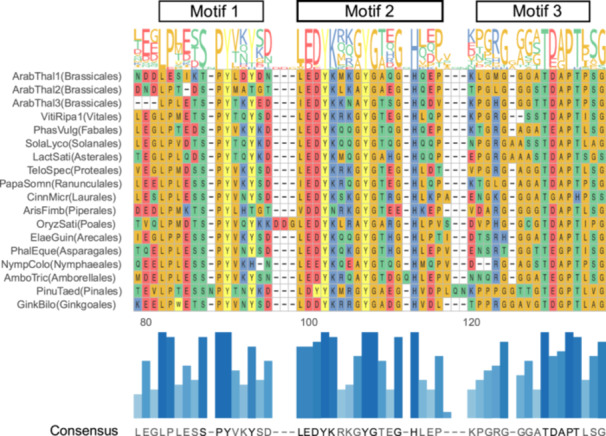
Multiple alignment of LEA6 proteins from various plant seed taxa showing the regions containing the conserved motifs. This alignment only includes the sequences of the conserved region found in most basal LEA6 protein identified in *G. biloba* and representatives from Amborellales and Nymphaeales (members of basal angiosperms), together with other LEA6 proteins of angiosperms. The upper part of the figure shows the distinctive motifs obtained by MEME analysis using all sequences as shown in Supporting Information S1: Figure S5. A representative consensus sequence and the corresponding amino acid abundance are shown at the bottom. A complete multiple alignment including all LEA6 sequences is shown in Supporting Information S1: Figure S5. [Color figure can be viewed at wileyonlinelibrary.com]

**Figure 3:**
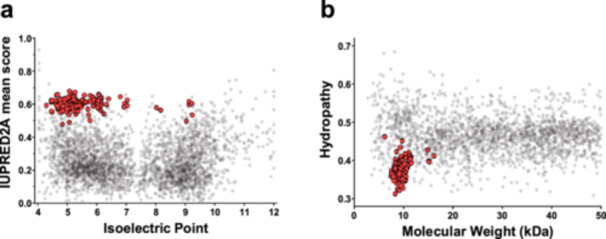
The structural disorder, isoelectric point, hydropathy, and molecular mass of 30,000 randomly selected proteins from *Arabidopsis thaliana* (grey dots) were compared against the plant LEA6 proteins (red dots) identified in this study. (a) The structural disorder of these proteins obtained by the IUPRED2A algorithm was plotted against their isoelectric point (pI). (b) The hydropathy of these proteins was plotted against their molecular mass. The values for pI and hydropathy of the proteins were obtained using the CIDER server. [Color figure can be viewed at wileyonlinelibrary.com]

The sequence alignment analysis of the 186 LEA6 proteins used for the phylogenetic study showed high similarity among them (mean similarity = 56%) (Supporting Information S1: Figure S5). We established three distinctive motifs in this family, some of which contain amino acid residues with a 90%–100% conservation (Figure 2 and Supporting Information S1: Figure S5). The high conservation of SPY and LEDYK sequences stands out, along with two additional tyrosine and one histidine residues in motifs 1 and 2, respectively, as well as the TDAPT sequence in motif 3. The two threonine residues in this last motif are potential phosphorylation targets (Mergner et al. 2020). Although the amino terminal region (1–33) of these proteins was previously considered a conserved motif (Battaglia et al. 2008), our analysis with a larger number of sequences did not show significant conservation (Supporting Information S1: Figure S5); nevertheless, we noticed a conserved enrichment of charged residues in this region (Supporting Information S1: Figure S6). Charged residues were found scattered along the rest of the LEA6 protein sequences, as well as small residues such as glycine and alanine (mean 20.1%). As it is common for other LEA proteins, we found low abundance of cysteine and tryptophan residues in this family, as fewer than five LEA6 sequences present these residues (Supporting Information S1: Figure S5). As expected, given the properties of the amino acids present in these proteins, the in‐silico analysis of their secondary structure showed a distinctive high structural disorder (Figure 3a). Likewise, most of LEA6 proteins (179/186) present an acidic isoelectric point (pI between 5 and 6), with lower values than the mean isoelectric point of Arabidopsis proteins reported as 6.81 (Kozlowski 2017) (Figure 3a). The molecular mass and the hydrophilicity of these proteins also distinguished LEA6 proteins from other Arabidopsis proteins (Figure 3b).

### 3.4 The LEA6 Protein Family in *A. thaliana*

**Figure 4:**
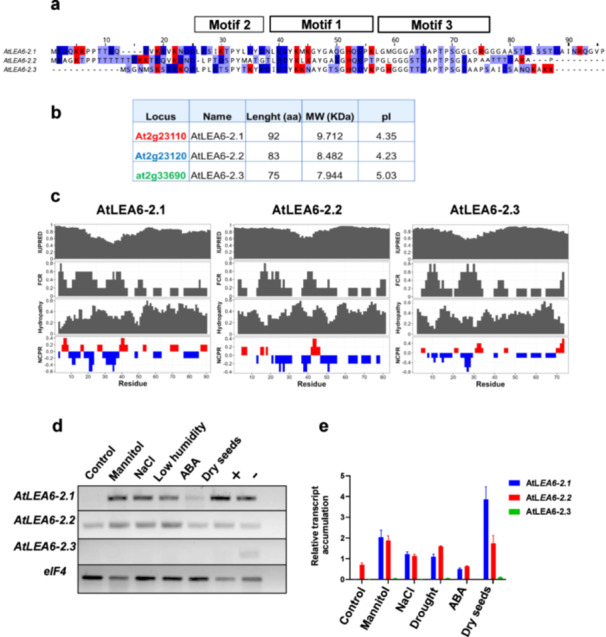
Sequence, physicochemical features, and transcript accumulation levels of Arabidopsis LEA6 proteins. (a) Multiple alignment of the amino acid sequences of the Arabidopsis LEA6 protein family. The alignment was colored using the Jalview (10.0.5) program to highlight the charge nature of their amino acid residues. Negative charged residues are indicated in blue, while those labeled in red correspond to the positive charged amino acid residues. (b) Amino acid number, molecular mass, and isoelectric point of Arabidopsis LEA6 protein family. (c) Graphs showing the net charge per residue (NCPR), the fraction of charged residues (FCR), the hydropathy, and the structural disorder (IUPRED) of AtLEA6 proteins. (d) Transcript accumulation patterns of Arabidopsis LEA6 family obtained by RT‐PCR. The growth conditions of the Arabidopsis seedlings from which the RNA was obtained is indicated in the upper part of this panel. Control: MS with no additions, Mannitol (300 mM), NaCl (200 mM), ABA (100 μM). For low humidity treatment, 7‐dag seedlings were transferred to a controlled humidity chamber (approx. 70% relative humidity) and collected after 24 h. Control corresponds to seedlings grown in MS medium under optimal conditions (see Materials and Methods). (−) RT‐PCR without DNA templates. (+) RT‐PCR using plasmid DNA containing AtLEA6 ORFs as templates. ABA, abscisic acid. (e) Transcript abundance was determined by densitometric analysis of agarose gel bands using ImageJ relative to *eIF4* transcript abundance. [Color figure can be viewed at wileyonlinelibrary.com]

*A. thaliana* has three *LEA6* genes located on chromosome 2, where *AtLEA6‐2.1* and *AtLEA6‐2.2* are positioned in tandem (Supporting Information S1: Figure S7). The proteins encoded by these genes are small with a molecular mass between 8 and 10 kDa, and with a predicted isoelectric point (pI) in the acid range between 4.23 and 5.03 (Figures 4a,b). An in‐silico analysis estimated that AtLEA6 proteins are highly hydrophilic and intrinsically disordered (Figure 4c). All AtLEA6 proteins show the family distinctive motifs, and a high percentage of negatively charged amino acid residues distributed along their sequence. Lysine is the most common positively charged amino acid residue in these proteins (Figures 4a,c).

Publicly available information reports that *AtLEA6‐2.1* and *AtLEA6‐2.2* transcripts are abundant in dry seeds (Klepikova et al. 2016), whereas *AtLEA6‐2.3* transcript is only found in mature pollen (Klepikova et al. 2015; Sullivan et al. 2019) (Supporting Information S1: Figure S8). Our RT‐PCR assays showed that *AtLEA6‐2.1* transcript was not detected under optimal growth conditions (Figure 4d–e). Clearly, it accumulates in dry seeds and in seedlings in response to high osmolarity (mannitol, NaCl), low humidity, and ABA treatments during vegetative growth (Figure 4d–e). In contrast, *AtLEA6‐2.2* transcript accumulates under optimal conditions in seedlings. This transcript was also detected in dry seeds and across all other tested conditions (Figure 4d–e). Notably, *AtLEA6‐2.3* transcript was exclusively detected in flowers, consistent with its accumulation in anthers and pollen as reported in the TraVa database (Klepikova et al. 2016; Sullivan et al. 2019) (Supporting Information S1: Figure S8a,b). This information is consistent with the presence in *AtLEA6‐2.1*, and *AtLEA6‐2.2* promoters of *cis*‐acting elements associated with the plant response to water limitation, such as ABRE, MYB, DRE, and MYC. Also, a *cis*‐element involved in seed expression (RY) was found in *AtLEA6‐2.1* promoter (Supporting Information S1: Figure S9).

### 3.5 Mutants Affecting the *AtLEA6‐2.1* Gene Are Sensitive to Water Deficit During Germination

**Figure 5:**
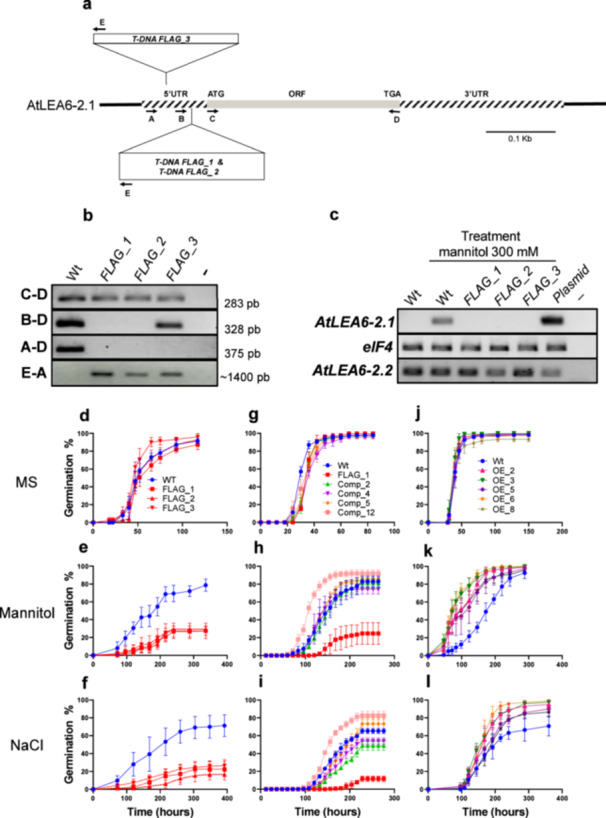
*atlea6‐2.1* mutants are sensitive during germination under high osmolarity. (a) Representative scheme of the *AtLEA6‐2.1* gene, indicating its corresponding transcript, including 5′ and 3′ UTRs (dashed boxes) and ORF (grey box). The localization of the oligonucleotides (arrows) and T‐DNA insertions are also shown. (b) PCR analysis to identify T‐DNA insertion sites and orientation in the different *atlea6‐2.1* mutants. (c) End‐point RT‐PCR analysis to determine *AtLEA6‐2.1* and *AtLEA6‐2.2* transcript accumulation levels in *atlea6‐2.1* mutants. *eIF4* was used as loading reference. Plasmid: RT‐PCR using as template plasmid DNA containing AtLEA6 ORFs. (−) RT‐PCR without DNA. (d–l) Germination rate of wild‐type (Wt) and *atlea6‐2.1* mutants. under optimal (d) and high osmolarity imposed with mannitol (e) and NaCl (f). Germination rate of complemented lines (g–i) and overexpressing (j–l) lines under optimal (g and j) and stress (h, k, i, and l) conditions. Germination was quantified by scoring radicle emergence using seeds from homozygous lines plated on MS medium (0.5×), or on MS added with mannitol (300 mM) or NaCl (200 mM). Seeds were stratified for 3 days and incubated in a growth chamber at 22°C. Error bars indicate SD of 3–5 replicates (*n* = 120–300), data were fit to a sigmoidal dose–response curve. Statistical analysis for germination rate is shown in Supporting Information S1: Figure S12. [Color figure can be viewed at wileyonlinelibrary.com]

To gain insight into the role of AtLEA6‐2.1 and AtLEA6‐2.2 proteins in the plant response to water deficit, we searched for mutants in the corresponding genes. We found three T‐DNA insertions affecting *AtLEA6‐2.1*, all of which are in its 5′‐UTR (FLAG_1, FLAG_2 and FLAG_3; Figure 5a). For the *AtLEA6‐2.2* gene, we found just one available T‐DNA insertion mutant localized in its ORF (ET9692) (Supporting Information S1: Figure S10a). The locations of the T‐DNA insertions were verified by PCR analysis (Figure 5b, Supporting Information S1: Figure S10b and Supporting Information S2: Table S3). The results confirmed that in all *AtLEA6‐2.1* mutants the T‐DNA insertions are in the 5′‐UTR (Figure 5a,b). Both FLAG_1 and FLAG_2 are in the same position, 21 bp upstream of the *ALEA6‐2.1* translation initiation site, while FLAG_3 is 68 bp upstream from this site (Figure 5a,b). The evaluation of *AtLEA6‐2.1* transcript levels, using oligonucleotide combinations spanning from the transcription start to the end sites, showed that the three T‐DNA insertions interrupt the gene transcript (Figure 5a,c). Because *AtLEA6‐2.1* and *AtLEA6‐2.2* genes are *in tandem*, we verified that *AtLEA6‐2.1* T‐DNA insertions were not affecting *AtLEA6‐2.2* transcript levels. RT‐PCR assays showed that *AtLEA6‐2.2* transcript abundance is not affected by the *AtLEA6‐2.1* T‐DNA insertions used here (Figure 5c).

These mutants were subjected to phenotypic characterization during germination in MS medium alone or containing either NaCl (200 mM) or mannitol (300 mM) (Figure 5d–l; Supporting Information S2: Table S6). Under non‐stress conditions, all *atlea6‐2.1* mutants showed similar germination rates and germination capacity (> 95%) as compared to the wild‐type line (Figure 5d). However, under high osmolarity (NaCl and mannitol), the reduction in germination rate and capacity was higher in the mutants than in wild‐type seeds (Figure 5e,f and Supporting Information S1: Figure S11). To determine whether the germination response was dependent on the stress intensity, mutant FLAG_1 was germinated in varying mannitol (150, 230 or 300 mM) (Supporting Information S1: Figure S12a–c) or NaCl (100, 150 or 200 mM) (Supporting Information S1: Figure S12d–f) concentrations. The results showed that the effect on the *atlea6‐2.1* germination efficiency was concentration dependent because germination efficiency gradually decreased as NaCl or mannitol concentrations increased (Supporting Information S1: Figure S12; Supporting Information S2: Table S7).

To confirm that the mutant phenotypes resulted from *AtLEA6‐2.1* malfunction, we performed complementation assays by expressing the *AtLEA6‐2.1* ORF under the control of the 35S promoter in the mutant backgrounds. The results showed that different lines expressing wild‐type *AtLEA6‐2.1* restored the wild‐type germination phenotype (Figure 5g–i) in the three mutant lines under high osmolarity (Figure 5h), demonstrating that the defective mutant phenotype was caused by an impaired *AtLEA6‐2.1* gene. Under 200 mM NaCl, the complementation lines exhibited a more variable germination phenotype that resembled that of the wild‐type (Figure 5i). This observation aligns with the enhanced germination seen under mannitol (Figure 5k) or NaCl (Figure 5l) when the AtLEA6‐2.1 protein was expressed in a wild‐type background (Figure 5j–l).

For *AtLEA6‐2.2*, the T‐DNA insertion disrupts the ORF region, 148 bp downstream of its ATG (Supporting Information S1: Figure S10b). Consistent with the T‐DNA location, RT‐PCR analysis showed that the T‐DNA insertion in the *atlea6‐2.2* mutant affects *AtLEA6‐2.2* transcript accumulation (Supporting Information S1: Figure S10c). In contrast to the stress‐sensitive phenotype of the *atlea6‐2.1* mutants, the *atlea6‐2.2* mutant line did not exhibit any detectable phenotype when germinated under high osmolarity imposed by NaCl (200 mM) or mannitol (300 mM) treatments (Supporting Information S1: Figure S10d,e and Supporting Information S2: Table S8).

### 3.6 Mutants Affecting the *AtLEA6‐2.1* Gene Showed Sensitivity to Water Deficit During Post‐Germination Growth

**Figure 6:**
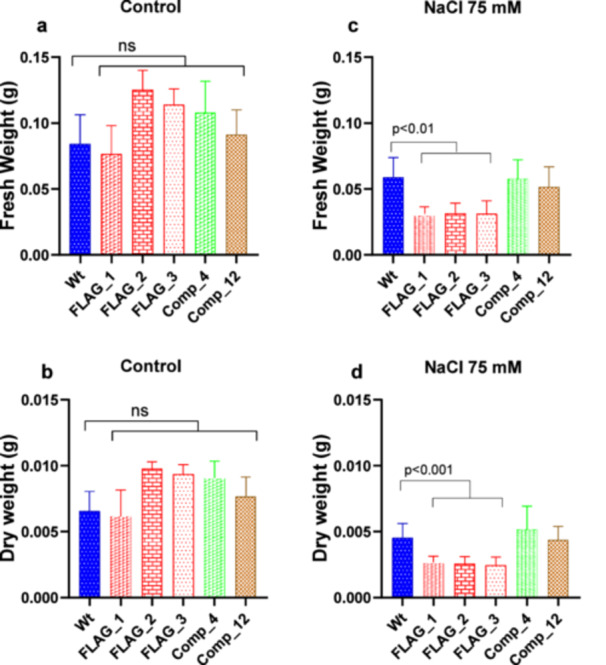
Seedlings (15 days after germination, dag) of *atlea6‐2.1* mutants lines show sensitivity under NaCl treatment (FLAG_1, FLAG_2, FLAG_3) as compared to wild‐type line (Wt), and they are complemented by the wild‐type gene (Comp_4 and Comp_12). Five dag seedlings were transplanted to control medium (a and c) or to MS supplemented with 75 mM NaCl (b and d). Fresh (a and b) and dry weight (c and d) were recorded from seedlings collected 15 days after transfer (dat). Error bars indicate SD of 3–5 replicates (*n* = 24). GraphPad was used to calculate One‐Way ANOVA. Tukey post hoc test was used to evaluate statistical significance. The *p*‐value of only significant different comparisons is shown. The values for the different lines in (a and b) did not show significant differences as compared to Wt. *p*‐value > 0.05 are statistically not significant (ns). [Color figure can be viewed at wileyonlinelibrary.com]

To evaluate whether the role of AtLEA6‐2.1 protein in response to water deficit extends to post‐germination stages, we transferred Arabidopsis seedlings (5 days) of the *atlea6‐2.1* mutant and wild‐type lines to media containing 75 mM NaCl. After 12 days of stress treatment, the mutant lines showed a significant reduction in fresh (Figure 6a,c and Supporting Information S2: Table S9) and dry weight (Figure 6b,d and Supporting Information S2: Table S9) when compared to wild‐type seedlings. We did not detect significant growth differences between the evaluated lines under non‐stress conditions (Figure 6a,b). Supporting the role of AtLEA6‐2.1 protein in the plant's tolerance to this stress condition, the expression of *AtLEA6‐2.1* gene in a mutant background complemented the salinity sensitive phenotype of mutant lines (Figure 6c,d).

### 3.7 AtLEA6‐2.1 Protein Is Essential for Proper Root Development Under Salt Stress

**Figure 7:**
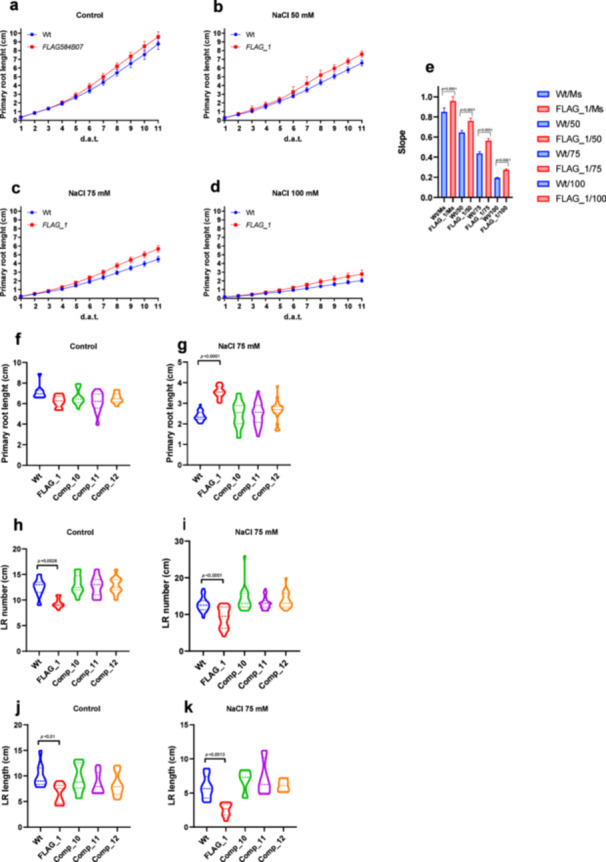
Primary root length kinetics of *AtLEA6‐2.1* wild‐type and mutant (FLAG_1) lines of Arabidopsis seedlings grown in MS (a) or MS supplemented with NaCl: 50 mM (b), NaCl 75 mM (c), or NaCl 100 mM (d). The primary root kinetics was followed for 11 days after transplanting (11 dat). *n* = 23–27. Analysis was performed with data from three independent experiments. (e) Statistical analysis for primary root growth rate rate. GraphPad was used to calculate One‐Way ANOVA. Tukey post hoc test was used to evaluate statistical significance. (f–g) Primary root length of wild‐type (Wt), *atlea6‐2.1* mutant (FLAG_1), and complemented (Comp_10, Comp_11, and Comp_13) seedlings grown under control (f) and NaCl conditions (75 mM) (g). Seedlings were sown in MS and transplanted at the second day to MS (f) or to MS supplemented with NaCl 75. mM (g). The primary root length was measured at 9 dat. (h and i) Lateral root (LR) number of seedlings transplanted to MS (h) or to MS supplemented with NaCl 75 mM (i). (j and k) Lateral root (LR) length of seedlings transplanted to MS (j) or to MS supplemented with NaCl 75 mM (k). Error bars indicate SD of six replicates (*n* = 24). GraphPad was used to calculate One‐Way ANOVA. Tukey post hoc test was used to evaluate statistical significance. See details in Section 2. *p*‐value > 0.05 are statistically not significant. The *p*‐value of only significant different comparisons are shown. [Color figure can be viewed at wileyonlinelibrary.com]

Given that *atlea6‐2.1* seedlings exhibited reduced growth under stress (Figure 6), we further investigated the effect of salinity on root development. For this, we measured primary root length, and number and length of lateral roots in 11 dat wild‐type and *atlea6‐2.1* seedlings under non‐stress and stress conditions. We first explored root growth sensitivity on MS media supplemented with 0, 50, 75 or 100 mM NaCl (Figure 7a–d; Supporting Information S2: Table S10). After germinating seedlings on salt‐free media, we transferred them to MS media with or without NaCl. Daily measurements of primary root length showed that under salinity conditions, *atlea6‐2.1* seedlings developed longer primary roots and higher growth rate compared to wild‐type (Figure 7a–e). This phenotype became more pronounced with increasing salt concentrations, indicating a direct association with the stress treatment (Figure 7a–e). To verify whether this phenotype resulted from AtLEA6‐2.1 deficiency, we obtained Arabidopsis lines expressing *AtLEA6‐2.1*, under the control of the 35S promoter, in the mutant background. The root phenotypic analysis of different independent lines did not show significant differences under control conditions as compared to the wild‐type (Figure 7f). However, when seedlings were grown in 75 mM NaCl media the root lengths were like those of wild‐type plants (Figure 7g), demonstrating the participation of AtLEA6‐2.1 protein in primary root development under stress.

Notably, *atlea6‐2.1* lateral root number was affected under non‐stress (Figure 7h) and stress conditions (Figure 7i). When exposed to salinity, lateral root number was significantly lower in the *atlea6‐2.1* mutant (FLAG_1) than in wild‐type lines (Figure 7i). Moreover, AtLEA6‐2.1 deficiency also altered the length of lateral roots under both growth conditions (Figure 7j,k). The number and length of lateral root showed by the *atlea6‐2.1* mutant was complemented in plants expressing the wild‐type LEA6 protein, supporting the requirement of this protein for a typical root development (Figure 7h–k; Supporting Information S2: Table S10).

### 3.8 AtLEA6‐2.1 Is Implicated in Seed Aging

**Figure 8:**
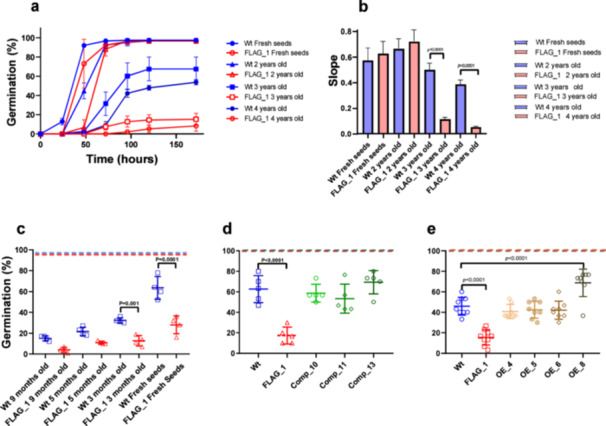
Seed longevity of wild‐type and *atlea6‐2.1* mutant. (a) Germination rate under. optimal conditions of wild‐type and *atlea6‐2.1* (FLAG_1) seeds stored during different periods in years, as indicated in the left legend. Each point corresponds to the mean germination of three independent replicates (*n* = 300). Error bars indicate SD. (b) Statistical analysis of germination rate using One‐Way ANOVA. Tukey post hoc test was used to evaluate statistical significance. (c) Accelerated aging test using seeds stored during different periods in months, as indicated at the bottom of the graph (*n* = 400), the upper dotted lines indicate the average final germination of both the wild‐type line (blue) and the mutant line (red) for all months. (d) Accelerated aging test of wild‐type, *atlea6‐2.1* (FLAG_1) and three independent complemented lines (*n* = 150). The upper dotted lines indicate the average final germination of the wild‐type (blue), mutant (red), and complemented (green) lines. (e) Accelerated aging test of wild‐type, *atlea6‐2.1* (FLAG_1) and three independent overexpression lines (*n* = 270–300). The upper dotted lines indicate the average final germination of the wild‐type (blue), mutant (red), and overexpression lines (brown). *p*‐value > 0.05 are statistically not significant. The *p*‐value of only significant different comparisons is shown. [Color figure can be viewed at wileyonlinelibrary.com]

Because AtLEA6.2‐1 accumulates in dry seeds, we explored the effect of its deficiency on seed viability. For this, we compared the germination rates and capacity under optimal conditions of Arabidopsis wild‐type and mutant seeds stored over 2, 3, and 4 years against recently harvested seeds. Both wild‐type and mutant seeds achieved nearly 100% germination and similar germination rates after 1 month or 2 years of storage (Figure 8a,b). Germination rates and capacity decreased after 3 years of storage and even more significantly after 4 years (Figure 8a,b). However, the reduction was much more pronounced in the mutant seeds −48% and 5% germination at three and 4 years, respectively—compared to wild‐type seeds, which showed 65% and 16% germination at the same time points (Figure 8a and Supporting Information S2: Table S11). This attribute was also explored using an accelerated seed aging assay to subsequently determine their germinating capacity in MS medium under standard conditions. As in the case of natural aging tests, accelerated aging assays using fresh seeds (1 month of storage) showed that *atlea6‐2.1* seeds present a lower germination capacity (30%) than wild‐type seeds (65%) (Supporting Information S2: Table S11). Notice that this assay has a stronger impact on seed germination than natural aging given the severity of the treatment. This was evident because the accelerated assay allowed to detect an impact on the germination capacity even in seeds stored for less than 1 year (Figure 8c). By conducting accelerated ageing assays, we showed that this mutant phenotype was complemented in independent lines expressing the wild‐type *AtLEA6‐2.1* gene (Figure 8d), demonstrating the participation of AtLEA6‐2.1 protein in the preservation of the plant embryo over time. When wild‐type *AtLEA6‐2.1* gene was expressed in a wild‐type background, only one line showed higher germination capacity than the wild‐type line, while the other three overexpression lines exhibited a germination percentage like wild‐type seeds (Figure 8e).

### 3.9 The Glassy Properties of Seeds Are Affected in *atlea6‐2.1* Mutants

**Figure 9:**
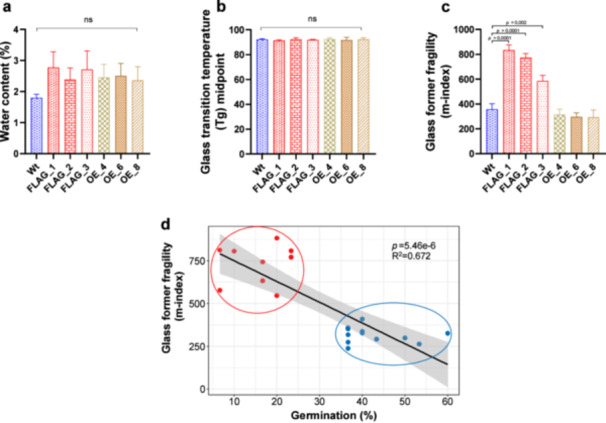
Glassy properties of seeds from wild‐type (Wt), mutant (FLAG_1, FLAG_2, FLAG_3), and overexpression lines (OE_4, OE_6, and OE_8). (a) Retained water content of different seed lines, (b) glass transition temperature (Tg) midpoint values, and (**c**) glass former fragility (m‐index) values. (d) Plot of correlation between glass former fragility (m‐index) and germination capacity. Statistics were calculated using a one‐way ANOVA and Tukey post hoc test: *p*‐value > 0.05 are statistically not significant. Significant statistically values are shown in bar charts, error bars represent standard error (SD) and 95% confidence intervals (CI) in correlation plots. ns: nonsignificant. [Color figure can be viewed at wileyonlinelibrary.com]

As the protection provided by the glassy state has largely been attributed to increased cytosolic viscosity upon drying (Buitink and Leprince 2008), we investigated whether the absence of AtLEA6‐2.1 affects the glassy properties of dry seeds. To begin this analysis, we measured the Tg of seeds from wild‐type plants, three *atlea6‐2.1* lines, and three AtLEA6‐2.1 overexpressing lines in a wild‐type background. A clear glass transition was observed in wild‐type seeds, with a midpoint of 92.197°C (Figure 9a and Supporting Information S2: Table S12). This transition was also present in *atlea6‐2.1* and in overexpression lines with no significant variation between them (Figure 9a). Although Tg midpoint is traditionally used to report Tg, the onset and endset temperatures of the glass transition can also provide relevant information, as they define the temperature range over which the glass transition occurs. Here, like the Tg midpoint, the onset and endset Tg temperatures were statistically indistinguishable among all lines examined (Supporting Information S1: Figure S13 and Supporting Information S2: Table S12). These data suggest that the presence or absence of AtLEA6‐2.1 does not influence the Tg of dry seeds.

We next examined the glass‐former fragility of these seeds. Glass‐former fragility is typically quantified by calculating a system's m‐index (see Section 2), where a high m‐index indicates a system with greater glass‐former fragility relative to a system with a lower m‐index (Crowley and Zografi 2001). Calculating the m‐index of wild‐type and *atlea6‐2.1* seeds showed that AtLEA6‐2.1 deficiency had a significant increase in glass‐former fragility relative to wild‐type seeds (Figure 9b and Supporting Information S2: Table S12), indicating that AtLEA6‐2.1 contributes to the formation of a more viscous and protective cellular environment in seeds. The overexpressing AtLEA6‐2.1 lines showed similar m‐indices to those obtained for wild‐type seeds. These results indicate that while the presence of AtLEA6‐2.1 do not affect the glassy properties of the seed once dry, it significantly increases the seed viscosity during the drying process compared to seeds lacking AtLEA6‐2.1 (Figure 9b).

To determine whether the influence of AtLEA6‐2.1 on the glassy properties of dry seeds is direct or indirect, we investigated how water content affects both Tg and glass‐former fragility as these properties are influenced by hydration levels. Increased water content generally lowers a system's Tg through a plasticization effect, where water acts as a plasticizer by reducing intermolecular interactions, enhancing molecular mobility and thereby decreasing Tg. This plasticization effect also tends to increase glass‐former fragility (Blasi et al. 2005; Eslami et al. 2023; Matveev 2000; Ramirez et al. 2024). Therefore, we aimed to determine if the observed increase in glass‐former fragility in *atlea6‐2.1* mutants was due to a direct effect of the protein or an indirect consequence of changes in water content. Using thermogravimetric analysis, we quantified water content in wild‐type, mutants, and overexpression seeds (Figure 9c and Supporting Information S2: Table S12). Water content was not significantly different between any of the lines tested (Figure 9c), nor was there a significant correlation between water content and either Tg or glass‐former fragility (Supporting Information S1: Figure S13c,d). This lack of correlation suggests that changes in glass‐former fragility are likely attributed to the presence or absence of AtLEA6‐2.1 rather than indirectly through water retention.

Among the different material parameters analyzed, only m‐index or glass former fragility varied between seeds containing or lacking AtLEA6‐2.1. All *atlea6‐2.1* lines exhibited a higher m‐index and lower germination capacity relative to wild‐type and overexpressing lines (Figure 9d and Supporting Information S2: Table S12). Furthermore, correlative analysis identified a significant inverse correlation between the m‐index (glass former fragility) and germination rate (Figure 9d). These findings underscore the contribution of AtLEA6‐2.1 in establishing a cellular environment during drying with sufficient viscosity to protect cellular components.

### 3.10 AtLEA6‐2.1 Shows Protective Activity During In Vitro Dehydration

**Figure 10:**
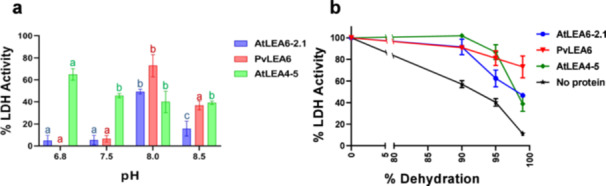
Protection dehydration assays using LEA6 proteins. (a) Protection capacity of different LEA proteins under different pH conditions during In Vitro gradual dehydration. Protection was determined at 97% after gradual dehydration. (b) Protection capacity of different LEA proteins under different dehydration levels at pH 8. In this case, the maximum achieved dehydration was 99%. LDH was used as a reporter enzyme. LEA6 proteins used in these assays are indicated in the right legends. 10:1 (LEA:LDH) monomer molar ratios were used in all cases. Error bars of all panels indicate SD. Data from these experiments were obtained from three independent replicates. In A, lower letters indicate statistically significant differences. [Color figure can be viewed at wileyonlinelibrary.com]

To explore potential molecular mechanisms for the function of AtLEA6‐2.1 protein, we performed In Vitro protection assays under gradual dehydration. Although a protective effect was not observed for the LEA6 protein from *P. vulgaris* (PvLEA6) (Reyes et al. 2005), we decided to investigate the In Vitro protective capacity of the Arabidopsis LEA 6 protein. The results showed a low protection effect (< 10%) when dehydration reached approximately 98% (pH 7.5), similar to what was previously reported for PvLEA6 protein (Figure 10a and Supporting Information S2: Table S13). This result contrasts with the higher protective activity observed for a LEA4 protein (AtLEA4‐5) (> 60%) (Figure 10a). The analysis of the physicochemical properties of LEA4 and LEA6 proteins showed that they differ in their isoelectric points. AtLEA6 proteins have an acidic pI (5.0–4.5) like PvLEA6 (6.0), whereas AtLEA4 proteins have a basic pI (10) (Supporting Information S1: Figure S14). This prompted us to perform these assays under different pH conditions during dehydration. Remarkably, the results showed that the two LEA6 proteins tested achieved a higher protecting effect under more basic pH conditions (7.8–8.5). AtLEA6‐2.1 and PvLEA6 proteins attained their highest protection effect at pH 8.0 (49% and 73%, respectively) (Figure 10a). Under this condition AtLEA4‐5 protein showed lower protection (40%) than at pH 6.8 (65%) (Figure 10). When the protective activity of these proteins was determined under different dehydration levels at pH 8.0, LEA6 and LEA4‐5 proteins showed similar protection activities (Figure 10b and Supporting Information S2: Table S13). Under the highest dehydration (approximately 99%), PvLEA6 showed higher protection (70%) than AtLEA6.2‐1 and AtLEA4‐5 (46% and 38%, respectively) (Figure 10b). These data indicate that the protective activity of each LEA protein is determined by its physicochemical properties and by those of the surrounding environment.

## 4 Discussion

The late embryogenesis abundant (LEA) proteins are strongly associated with desiccation tolerance due to their accumulation during seed maturation when dehydration begins (Hernández‐Sánchez et al. 2022). They are thought to protect the embryo from damage during desiccation and rehydration. A strong correlation exists between increased LEA protein transcript and protein levels in vegetative tissues when plants are subjected to water deficit conditions (Battaglia et al. 2008; Hernández‐Sánchez et al. 2022; Hundertmark and Hincha 2008). These observations, coupled with the sensitivity to water deficit in deficient mutants of certain *LEA* genes and the tolerance conferred by overexpression of LEA proteins, suggest a key role for these proteins in the ability of plants to withstand water scarcity. However, although LEA proteins are abundant in dry seeds, it is noteworthy that most LEA groups emerged in evolution long before the advent of seeds (Becker et al. 2020). Group 6 LEA proteins are a notable exception to this trend. This study leverages data from many sequenced genomes, including those of early‐evolved seed plants, to establish that the LEA 6 protein family originated concurrently with the appearance of seeds. This finding is supported by the presence of LEA6 proteins in gymnosperms like Ginkgo and Pinus, and their absence in ferns (Marchant et al. 2022). Of note, Group 6 LEA proteins were not found in the Ceratophyllales sequenced genomes, a group of flowering plants that primarily live in submerged aquatic environments. These observations strongly suggest LEA 6 proteins are involved in seed desiccation tolerance and viability over time, hallmark features of orthodox seeds. The association of LEA 6 proteins with these characteristics underscores their role in seed resilience and longevity, which is crucial for the preservation and successful germination of orthodox seeds.

The phylogenetic analysis performed in this study led us to suggest that most existing proteins derive from the original LEA6 protein or from one of three key ancestral duplications. This agrees with the synteny analysis reported by Artur et al. (2019), indicating lineage‐specific duplications for LEA6 family. These ancient duplications affected only 10 out of the 27 orders studied, with the remaining orders, including all five monocot orders examined, showing no evidence of such duplications. At lower taxonomic levels, duplications and losses are common. This dynamism has significantly shaped the evolution of the LEA6 family.

The relatively short evolutionary history of LEA6 proteins may explain the high degree of conservation among family members. Despite substantial gene losses throughout plant evolution, LEA6 proteins exhibit remarkably conserved motifs (Battaglia et al. 2008). This study expanded on previous analysis by examining a larger sequence data set and confirmed the presence of three previously documented motifs. The SPY‐‐Y‐‐LEDYK‐‐‐YGT motif stands out with over 90% conservation, with the tyrosine residues particularly well preserved. The histidine residue in the GGT‐GH‐‐P motif is 100% conserved across all sequences, hinting at a potential metal binding ability (Cragnell et al. 2019; French‐Pacheco et al. 2018). Furthermore, we identified potential phosphorylation sites in the conserved TDAPT‐SG motif, as supported by phosphoproteomic data and prediction information (Mergner et al. 2020). Phosphorylation could therefore be a mechanism for modulating LEA6 protein function. The conservation of multiple proline residues across LEA6 protein sequences is also notable, suggesting potential bending sites that could induce folding upon local changes in charge distribution (Williamson 1994). The highly conserved motifs in this family highlight their biological significance, potentially as interaction sites with client proteins, and/or as regions required for specific conformation(s) under certain conditions.

LEA6 proteins, like other LEA proteins, have the physicochemical properties of intrinsically disordered proteins (IDPs), resulting in the absence of a stable secondary structure and the presence of a multiple conformations due to increase exposure of amino acid residues to the solvent. This structural disorder makes them sensitive to their surrounding environment and allows fuzziness, a disorder‐to‐order transition influenced by intra‐ and inter‐molecular interactions, according to their mean net charge (valence), and charge distribution (Holehouse and Kragelund 2023; Oldfield and Dunker 2014; Tompa and Fersht 2009; Wright and Dyson 2015). The acidic pI of most LEA6 proteins indicates a high proportion of negatively charged amino acid residues. In many cases, clusters of negatively charged motifs are separated by few residues with opposite charge suggestive of an array of interaction motifs, containing distinctive amino acid residues.

Unlike other species, where the LEA6 family has a single member, Brassicaceae family has experienced multiple duplication events. In *A. thaliana*, this family consists of three members, all of them showing an acidic pI (4.2–5.0) and low molecular mass (7.9–9.7 kDa). Transcript abundance analyses suggest that these proteins may be required under different environmental conditions, in different plant tissue, and/or at different developmental stages. Notably, *AtLEA6‐2.3* transcript accumulates in pollen grains (ATHENA) (Klepikova et al. 2015), a plant structure that, like orthodox seeds, undergoes obligatory dehydration during development. The high conservation among LEA6 family members suggests they may perform similar functions in different tissues at specific developmental stages or under certain environmental conditions. This idea is supported by the diversification of their regulatory regions, evidenced by the presence of distinct cis‐elements in their promoters (Supporting Information S1: Figure S9), which might contribute to the observed transcript accumulation patterns, further highlighting the nuanced regulatory mechanisms controlling LEA6 protein activity.

This study focused on two *LEA6* genes, *AtLEA6‐2*.1 and *AtLEA6‐2.2*, which are expressed in seeds and during vegetative growth in response to water deficit. However, only *atlea6‐2.1* mutant lines display sensitivity to high osmolarity (300 mM mannitol) and/or salinity (200 mM NaCl). The AtLEA6‐2.1 deficiency affects seed germination rate and capacity, as well as post‐germination seedling growth under stress, demonstrating this protein is required for optimal plant adjustment to these conditions. Complementation of this phenotype by the wild‐type *AtLEA6‐2.1* coding sequence supports this conclusion. In contrast, *atlea6‐2.2* insertion mutant germinated like wild‐type seeds under stress. The difference in phenotype is consistent with their contrasting transcript abundance of the two genes in dry seeds: AtLEA6‐2.1 transcript level is higher than that of AtLEA6‐2.2. The high abundance of AtLEA6‐2.1 in seeds may allow this protein to persist during germination, helping to protect against damage caused by stressful environments. These findings suggest that, even though the *AtLEA6‐2.2* responds to salinity, its low abundance in seeds and its responsiveness to stress alone are not sufficient to provide protection against these conditions, at least, during these developmental stages. However, further studies under different conditions, developmental stages, and levels of analysis (e.g., tissue, cellular, molecular) are needed to uncover its function.

The sensitivity to low water availability conditions showed by AtLEA6‐2.1 deficiency adds to the related phenotypes reported for knockdown or knockout mutants in *LEA* genes from other families (Hernández‐Sánchez et al. 2022). For instance, knockout mutants in Arabidopsis *LEA4* (LEA_1) (Olvera‐Carrillo et al. 2010), and *ERD14* (dehydrin) (Nguyen et al. 2020), as well as in *Physcomitrella patens PpDHNAs* and *PpDHNB* (dehydrins) (Ruibal et al. 2012) have shown similar responses. Additionally, knockdown mutants have been reported in Arabidopsis and *Brassica napus*, *AtLEA3* and *BnLEA3*, respectively (Liang et al. 2019), in *Medicago truncatula CAS31* (dehydrin) (Li et al. 2020), and *Capsicum annum CaDHN5* (dehydrin) (Luo et al. 2019) and *CaDIL1* (LEA5) (Lim et al. 2018). Although these phenotypes have not all been characterized under the same conditions or developmental stages, they collectively highlight the requirement of LEA proteins from different families for an effective plant response to water limitation.

Despite the high conservation among LEA6 family members, these results indicate a lack of functional redundancy. This can be explained by their divergent expression profiles, resulting from the presence of different cis‐elements in their promoters. This finding highlights the temporal, spatial, and developmental specialization that each member of this family, and likely other LEA families, as suggested by the data referenced above, have acquired over evolution to optimize plant responses to environmental stress.

Unexpectedly, AtLEA6‐2.1 deficiency also impacted root architecture. Under salinity *atlea6‐2.1* mutant seedlings exhibited longer of primary roots than wild‐type seedlings, indicating that AtLEA6‐2.1 plays a role in controlling primary root length in response to stress. Of note, AtLEA6‐2.1 deficiency affects both the number and length of lateral roots, not only under salinity but also under non‐stress conditions, suggesting its role in lateral root development during both stress and non‐stress environments. This observation is consistent with the high *PvLEA6* gene expression in emerging lateral roots (Moreno‐Fonseca and Covarrubias 2001). While the mechanism by which LEA6 proteins contribute to root development is currently unclear, it is conceivable that this function is a consequence of their interaction with key regulators of these developmental processes, likely responding to changes in the cellular osmotic status.

Producing robust seeds is essential for improving crop yield. Vigorous seeds are characterized by efficient germination, strong seedling growth, even under stressful conditions, and the ability to maintain these characteristics after storage. Seed longevity, the capacity to maintain viability during prolonged storage of dry seeds, is a key determinant of seed vigor. Seeds gradually acquire longevity during maturation, reaching their peak near their dehydrated state (Leprince et al. 2017). LEA proteins are considered markers of late seed maturation, and their abundance has been correlated with increased longevity. However, the specific roles of LEA proteins in seed longevity remain largely unknown. This study investigated the role of AtLEA6‐2.1 in seed longevity and found that this protein is essential for this process. This conclusion is based on a comparison of *atlea6‐2.1* and wild‐type seeds stored under identical conditions for different lengths of time (0–4 years). The *atlea6‐2.1* seeds had significantly lower germination capacity compared to their wild‐type counterparts, highlighting the direct impact of AtLEA6‐2.1 on seed lifespan. Accelerated aging assays, partially simulating natural aging conditions (Hay et al. 2019; Rajjou et al. 2008), supported these observations, with mutant seeds consistently showing lower germination rates than wild‐type, underscoring the critical role of AtLEA6‐2.1 in maintaining seed viability. Acquisition of seed longevity is a complex trait, arising from the interaction of many factors that modulate different metabolic pathways and regulatory networks during seed development (Leprince et al. 2017). Therefore, AtLEA6‐2.1 is likely not the only factor involved in maintaining seed longevity. Previous reports showed that knockdown mutants of two Arabidopsis dehydrins, XERO1 and RAB18, had slightly reduced germination percentages after accelerated aging, suggesting their involvement in seed longevity (Hundertmark et al. 2011).

Vitrification, the formation of a glass‐like solid during desiccation, is a common strategy for surviving dehydration in many organisms across all kingdoms of life. This process reduces molecular movement by increasing viscosity, effectively halting metabolic activity during drying and reducing molecular motion to prevent damage to biological material (Boothby 2021; Boothby and Pielak 2017; Crowe et al. 1998; Sakurai et al. 2008). Orthodox dry seeds exist in a glassy or vitrified state, and this high viscosity may help to mitigate deterioration during storage, preserving embryo viability for extended periods. In Vitro studies using peptides from Group 3 LEA proteins (Pfam_4) suggested that these LEA proteins can form a stable glassy matrix in a dry state (Shimizu et al. 2010). This suggests that LEA protein' ability to modulate the glassy properties of cytoplasm in different cell types may contribute to the acquisition of seed longevity.

This study demonstrates that AtLEA6‐2.1 deficiency does not prevent glassy state formation in dry *A. thaliana* seeds, but it does alter their glass‐former fragility. This suggests that AtLEA6‐2.1 does not drive vitrification in seeds but rather modifies the properties of the resulting vitrified state. Glassy properties, including glass‐former fragility, contribute to the protection and stabilization of labile biomolecules within the vitrified system (Boothby 2021; Boothby and Pielak 2017; Boothby et al. 2017; Crowe et al. 1998; Kumara et al. 2024; Ramirez et al. 2024; Sakurai et al. 2008). To understand how glassy properties contribute to protection, this study examined Tg, glass‐former fragility, and water retention, in different AtLEA6‐2.1 seed lines. Mutant lines showed higher glass‐former fragility, suggesting more vulnerability to molecular movement and structural changes, consistent with their lower germination over time. This could be explained because during germination, when seeds rehydrate, a more stable vitrified state helps preserve critical proteins and cellular components, increasing the chances of successful germination by minimizing damage or loss of function (Sun and Leopold 1997; Zamecnik et al. 2021). Lowering glass‐former fragility makes the vitrified state more stable and protective (Ramirez et al. 2024; Wowk 2010) boosting dormancy, longevity and improving germination when conditions become favorable. While the mechanism by which AtLEA6‐2.1 improves germination and longevity is not fully understood, several possibilities exist. This protein may directly stabilize the glassy state by interacting with biomolecules and cellular structures, controlling molecular movement, and influencing water retention, all of which reduce fragility. The observation that some LEA proteins form oligomers and may organize into biocondensates under low water availability (Cuevas‐Velazquez et al. 2021; Hernández‐Sánchez et al. 2022), suggests they could adopt structural ensembles during dehydration, between themselves and/or with other cellular components, potentially modifying the biophysical properties of the intracellular environment. Additionally, AtLEA6‐2.1 might indirectly reduce fragility by enhancing the stress response through interactions with other proteins or signaling pathways (Hincha et al. 2021; Kc et al. 2024). These actions could collectively contribute to reduced fragility, increased stability, and enhance survival during desiccation. To our knowledge, this is the first study to demonstrate that the loss of a single protein can modify vitrified properties, directly linking it to protection in the dry state.

Many LEA proteins have demonstrated protective capabilities under In Vitro water‐limited conditions (reviewed by Battaglia et al. 2008). This study investigated the In Vitro function of LEA6 proteins using a progressive dehydration assay. Previous work showed that the LEA6 protein from *P. vulgaris* did not protect reporter enzymes in this assay, unlike LEA proteins from other families (Reyes et al. 2005). Given that most LEA6 proteins, including AtLEA‐2.1, have an acidic pI (4–6.5), this study hypothesized that the net charge and charge distribution in LEA proteins could influence their conformation and function under different pH conditions (Lyons et al. 2023). Therefore the In Vitro dehydration assays were conducted at various pH values. This study focused on recombinant AtLEA6‐2.1 protein due to its demonstrated role in the plant response to water deficit. We also included PvLEA6 (pI 6.5) because of its lack of protective activity in previous experiments (Reyes et al. 2005), and AtLEA4‐5, a Group 4 LEA protein with a basic pI (9.4). Typically, the protein mixture used in these In Vitro dehydration assays is buffered at pH 7.5. After dehydration, proteins are rehydrated in a buffer optimal for reporter enzyme activity. As expected, LEA6 proteins did not protect the reporter enzyme (LDH) under these conditions, consistent with previous results for PvLEA6 (Reyes et al. 2005). Remarkably, LEA6 proteins showed significant protective effects when dehydration was conducted at a pH above 7.5. This finding highlights the importance of considering the charged nature of structurally flexible LEA proteins, which can adopt different conformations and exhibit altered intra‐ and intermolecular association properties depending on the surrounding environment, including pH, ions, and water molecules. At a pH above their pI, LEA6 proteins have a net negative charge. The charge changes that occur between pH 7.5 and 8.0 may promote different hydrogen bonds and electrostatic interactions with client proteins. Bioinformatic analysis predicts only minor differences in the solvent‐exposed residues of AtLEA6‐2.1 and LDH between these two pH values (Supporting Information S1: Figure S15a). However, this study's data suggest that these subtle changes may be responsible for the differential protective activity of LEA6 proteins. In‐silico docking predictions support this possibility, indicating that at pH 8.0, the AtLEA6‐2.1 protein binds to a different region of LDH than it does at pH 7.5 (Supporting Information S1: Figure S15b,c). At pH 8.0, the AtLEA6‐2.1 binding region appears closer to the active site of LDH (Supporting Information S1: Figure S15c), consistent with the preservation of the LDH activity under dehydration. An additional finding from the dehydration assays was that the protective effect of both LEA proteins decreased at pH values above 8.0. Analysis of the predicted physicochemical changes in LDH under different pH conditions revealed an increase in enthalpy at higher pH values (Supporting Information S1: Figure S14d). This suggests that increasing pH might destabilized interactions required for maintaining LDH's active structure consistent with the low LDH activity recovery and the enzyme's sensitivity to high pH (Robin et al. 2023).

This study provides a comprehensive functional characterization of Arabidopsis Group 6 LEA proteins, showing that AtLEA6‐2.1 is essential for the plant's appropriate adjustment to water deficit and its deficiency alters Arabidopsis root development under salinity. We found that AtLEA6‐2.1 is required to maintain seed viability over time and to influence seed glassy properties. These findings highlight the importance of LEA6 proteins in plant responses to environmental changes. In Vitro assays revealed that AtLEA6‐2.1 exhibits chaperone‐like activity, protecting LDH from inactivation during dehydration at pH 8.0 but not at the commonly used pH of 7.5. This observation emphasizes the importance of considering both the physicochemical properties of LEA proteins and their surrounding environment to understand their function and identified potential targets. This information enhances our understanding of plant responses to stressful environments and provides insights for improving plant tolerance to water scarcity.

## Conflicts of Interest

The authors declare no conflicts of interest.

## Supporting information

Supplementary Figures Arroyo‐Mosso et al.

Supplementary Tables Arroyo‐Mosso et al.

supmat.

## Data Availability

The data that supports the findings of this study are available in the supporting material of this article.
