# Supplementary material for: A Group 6 LEA Protein Plays Key Roles in Tolerance to Water Deficit, and in Maintaining the Glassy State and Longevity of Seeds
Source: Plant Cell Environ. 2025 Jun 5;48(9):6874–96. doi: 10.1111/pce.15649 (PMC12319291; doi:10.1111/pce.15649)

Figure S1. Phylogeny of the LEA6 family showing paralogous proteins. The outer circles display paralogues, with each species represented in a distinct color, and a dot for each of the 14 large orders that exhibited duplications. Proteins without paralogues are shown in light grey. Other features are as described in Figure 1. This figure has the sufficient resolution to support zoomed viewing.

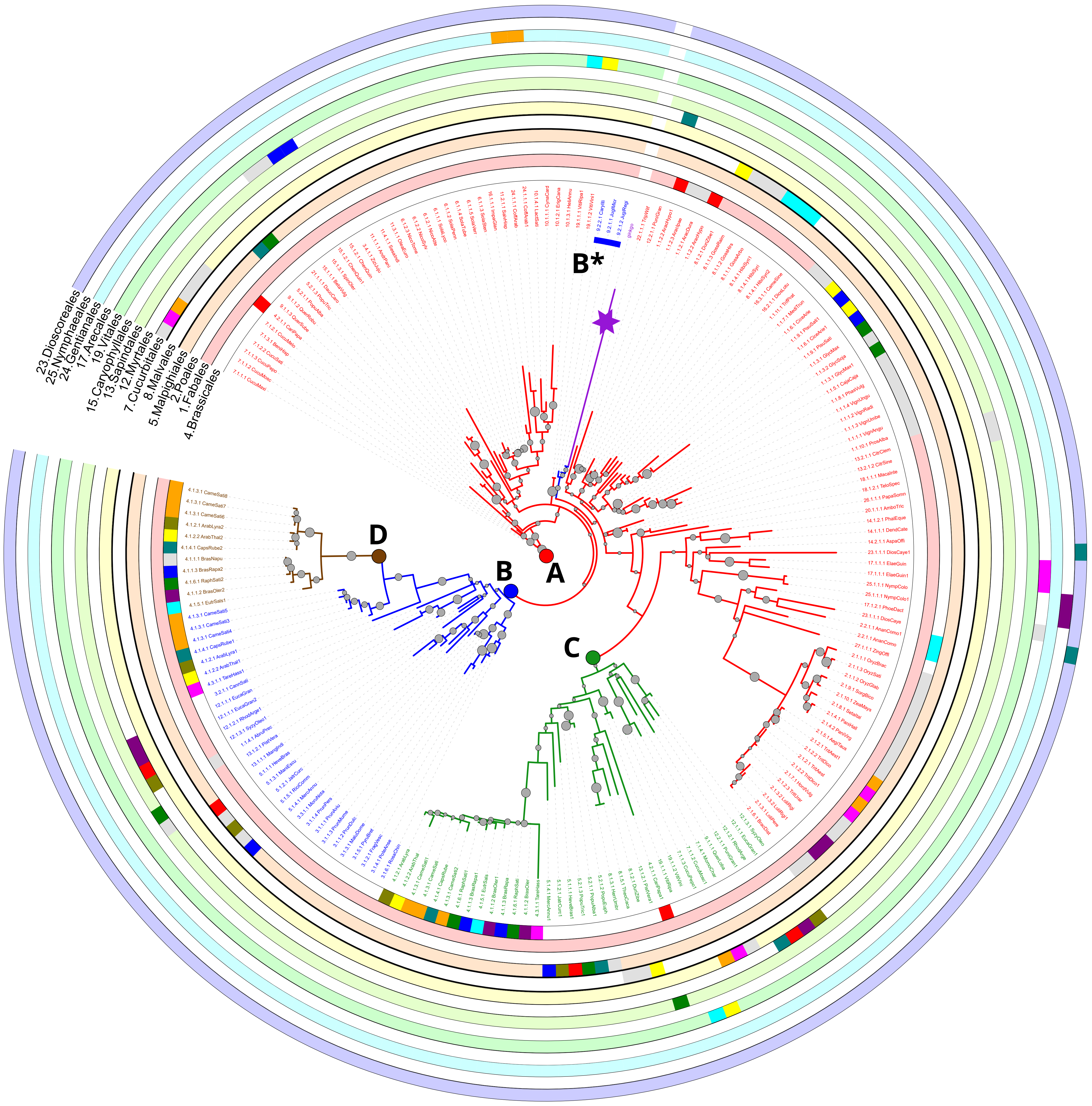

Figure S2. Reconciled tree of the LEA6 proteins with the species tree carried out using Treerecs. The species tree was derived from their NCBI taxonomy, with polytomies resolved randomly via PhyloT. This reconciliation reveals 45 duplications and 123 losses, resulting in a total reconciliation cost of 213. Species names are included the end of each branch. This figure has the sufficient resolution to support zoomed viewing.

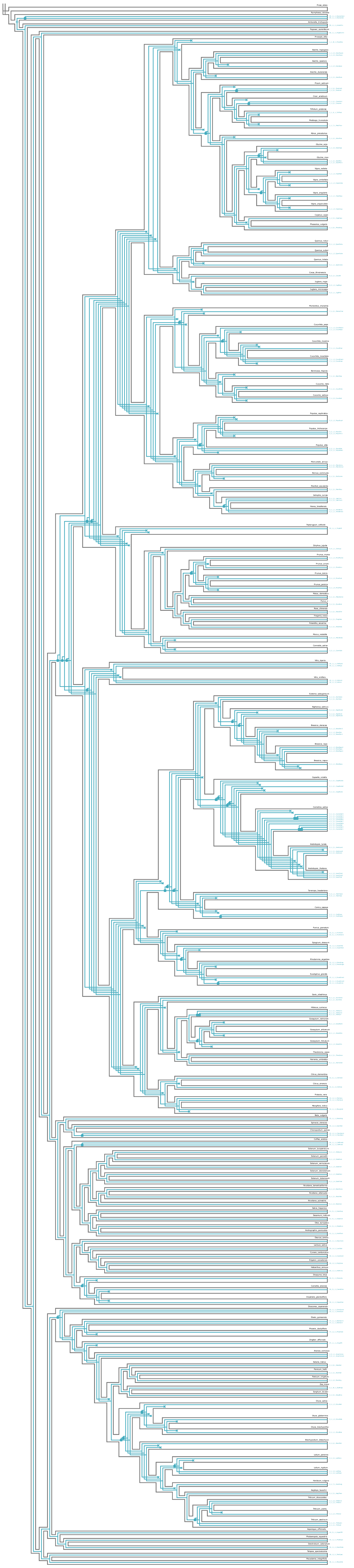

Figure S3. Reconciled tree of the LEA6 proteins with the species tree carried out using SpeciesRax. Unlike typical methods, SpeciesRax independently inferred the species tree using the MiniNJ method. The final image depicting duplication,loss,horizontal transfer, and speciation events,was generated by Thirdkind, a tool for creating graphical output from recphyloXML files. The evolutionary history shown is complex, involving 197 speciation events (including 19 speciations where one lineage went extinct), 18 gene duplications, and 19 horizontal transfer events. Our model, positing three ancestral duplications as explained in Figure 1, suggests a simpler evolutionary history. This figure has the sufficient resolution to support zoomed viewing.

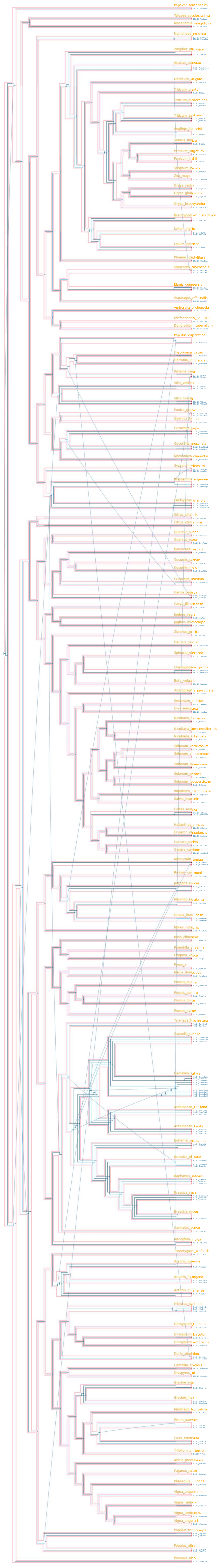

Figure S4. Phylogeny of the LEA6 family members from the order Brassicales, among the large orders, the one with most species and members. The inner color strip indicates the taxonomic species. The outer color strip denotes the duplication event that originated the protein, according to our model postulated in Fig. 1. Labels and colors are as in Fig. 1. While most branches in the global phylogeny have poor bootstrap support, branches in the order level phylogenies (such as this one) showed a better support. However, the order level phylogenies agree broadly with topology of the global tree. In this tree, 4.2.1.1_CariPapa (*Carica papaya*) is the only member that does not group with other members. This agrees with its position in Fig. 1 as the sole representant of duplication A from the Brassicales. The other three duplications show high consistency with the global tree.

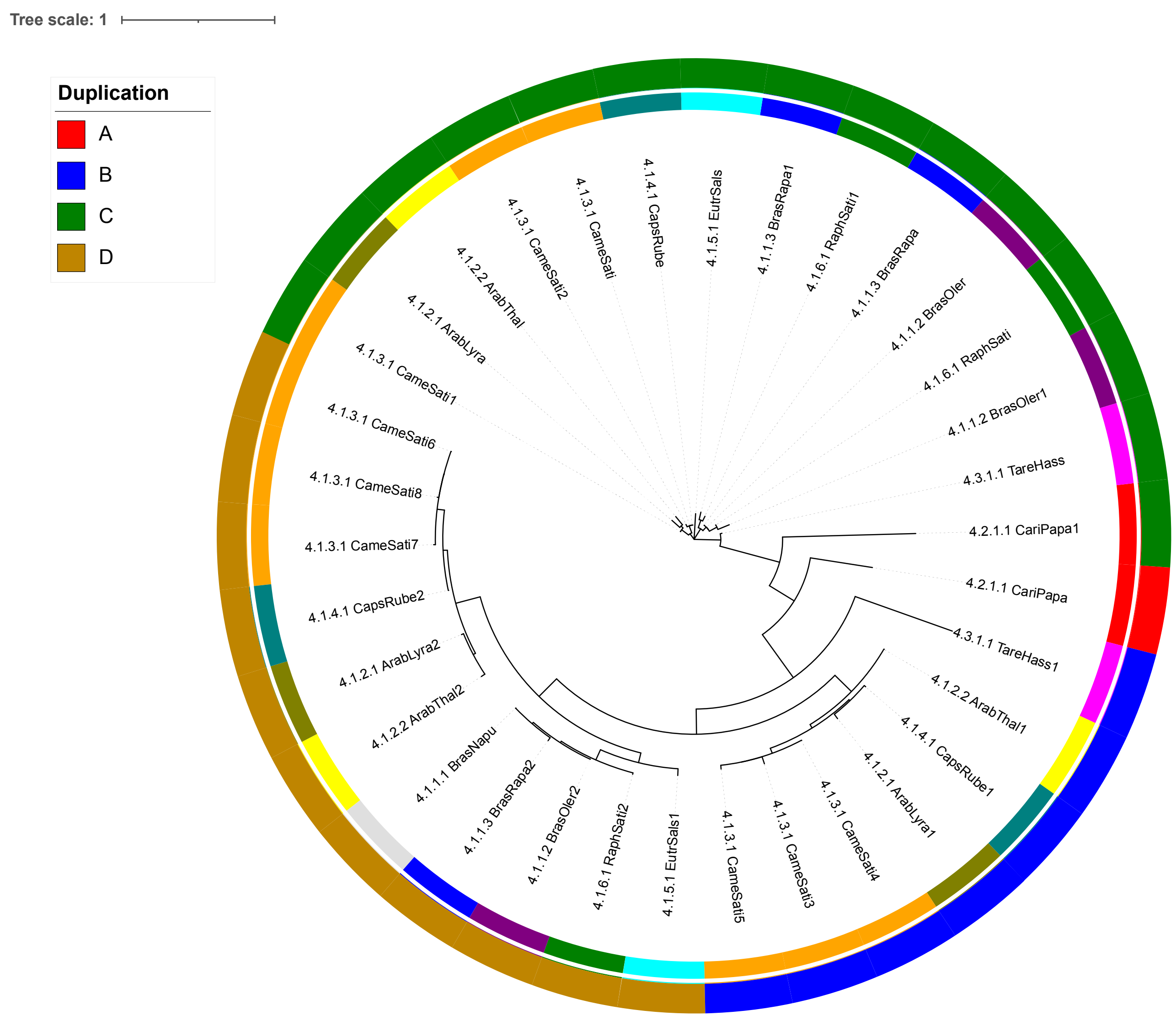

Figure S5. Multiple sequence alignment of 90 sequences of LEA6 proteins. The intensity of blue coloration represents the degree of conservation. Graph showing the conservation level of amino acid residues is presented at the bottom of the figure. This figure has the sufficient resolution to support zoomed viewing.

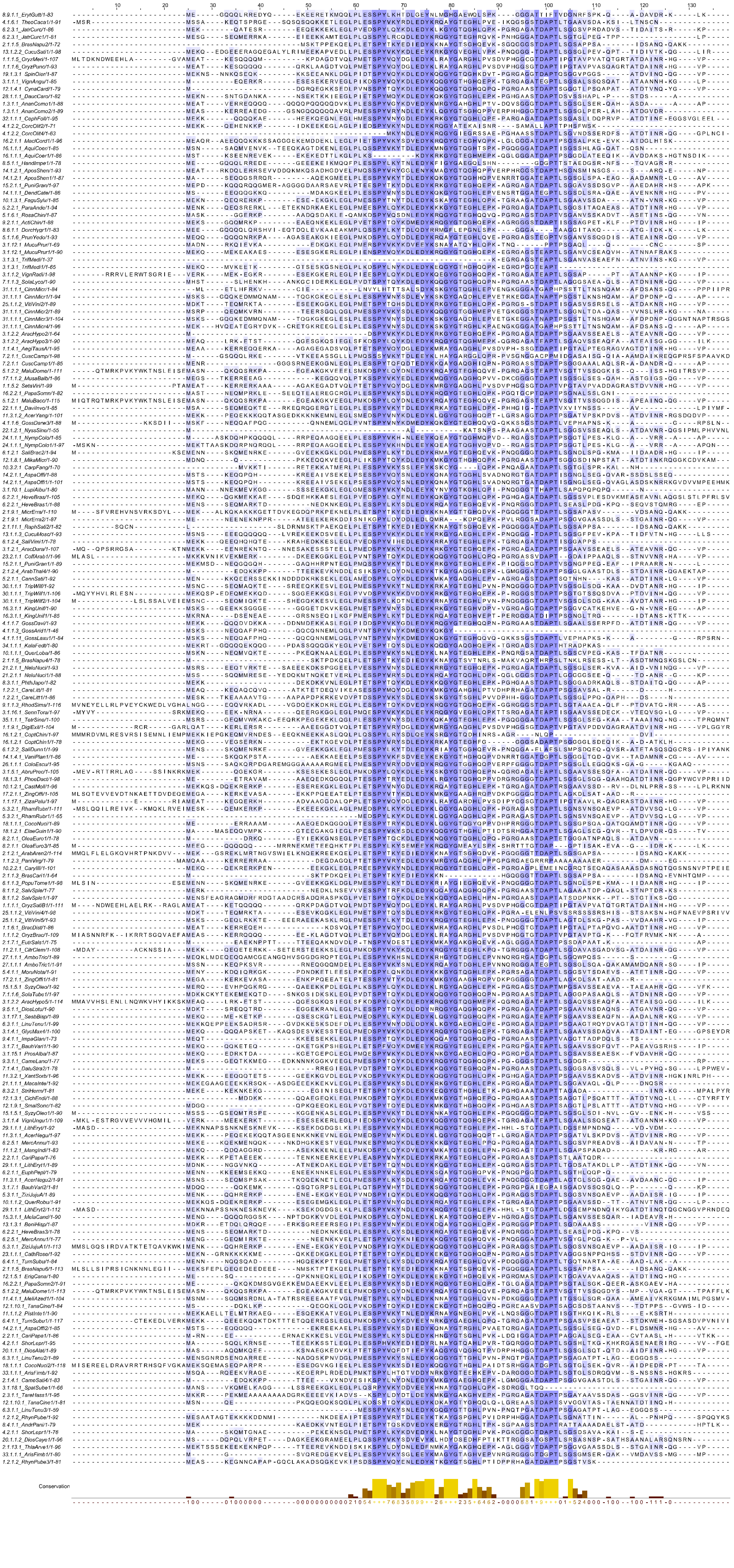
Conservation

Figure S6. Multiple sequence alignment of 90 sequences of LEA6 proteins. Positively and negatively charged residues are highlighted in red and blue, respectively. Green indicates amino acid residue with polar uncharged side chains. The bottom graph indicates the conservation level. This figure has the sufficient resolution to support zoomed viewing.

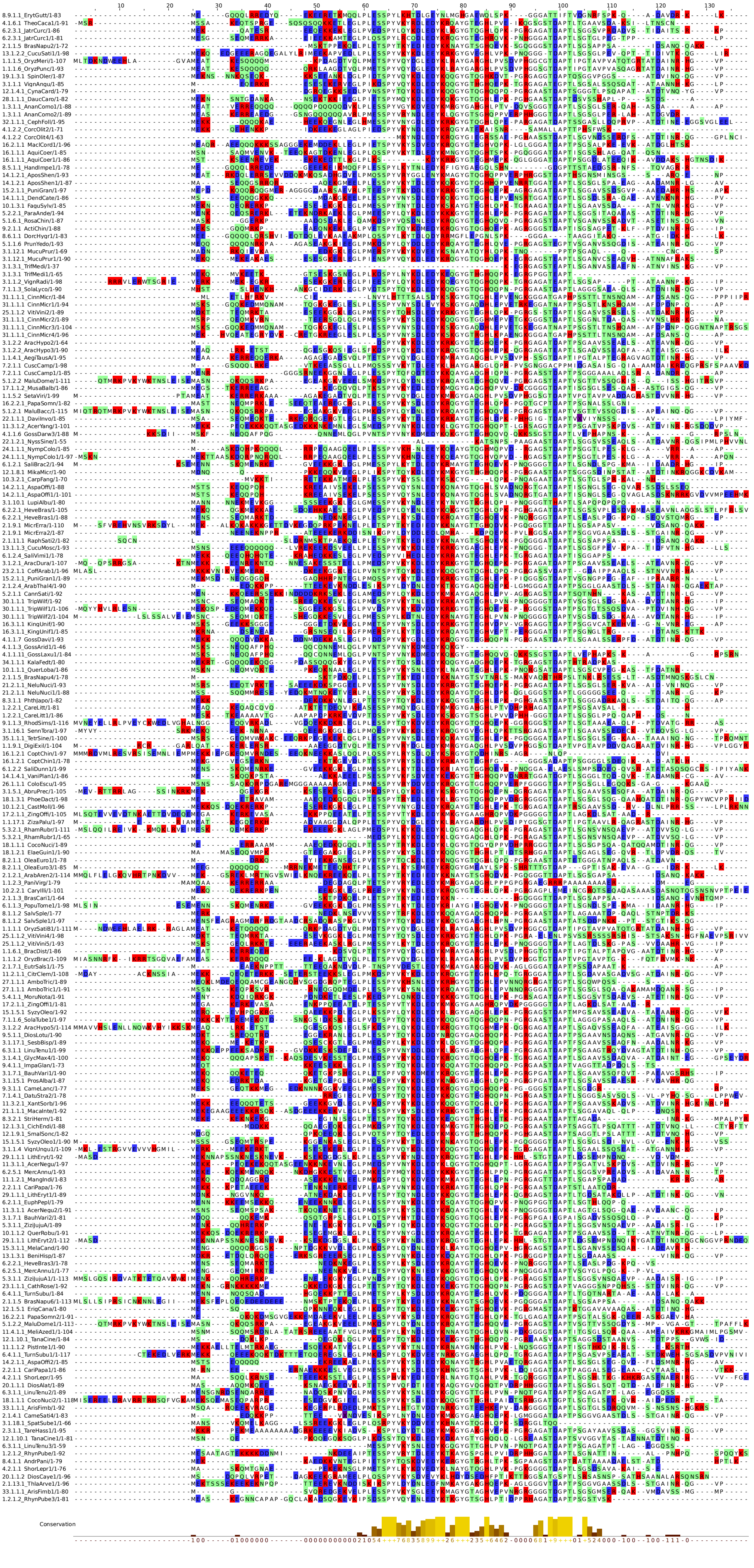
Conservation

Figure S7. Representative image of Arabidopsis chromosome 2, according to TAIR database, showing the approximate positions of *AtLEA6-2.1*, *AtLEA6-2.2*, and *AtLEA6-2.3* genes.

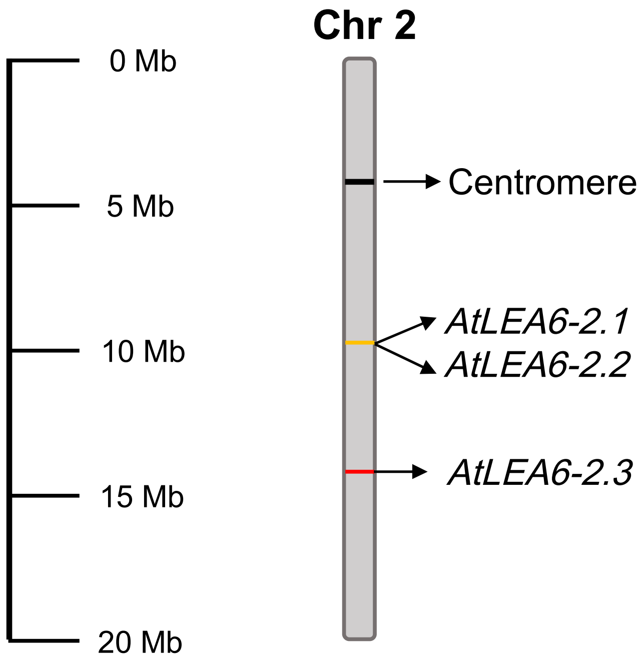

Figure S8. (**a**) Relative abundance of Arabidopsis LEA6 transcripts across different organs, as determined from transcriptomic data available in the TraVA database (Klepicova et al., 2016). (**b**) Transcript accumulation levels of AtLEA6 transcripts obtained by end-point RT-PCR using RNA from Arabidopsis flowers. (-) RT-PCRs without DNA template. (+) RT-PCRs using as templates plasmid DNA containing the respective AtLEA6 ORFs.

a

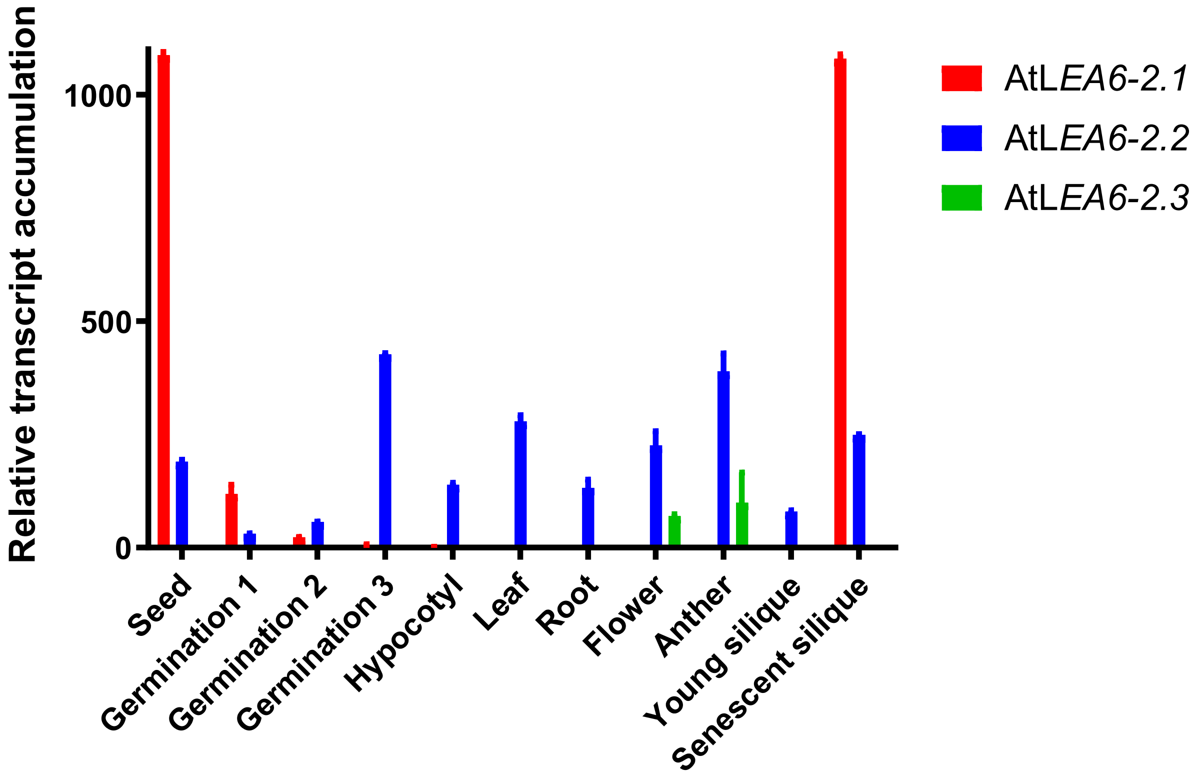

b

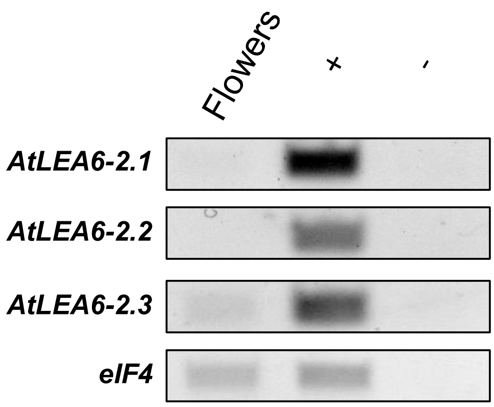

Figure S9. *cis*-elements in the promoter regions of *AtLEA6-2.1* (a), *AtLEA6-2.2* (b), and *AtLEA6-2.3* (c) obtained from PLACE database. Numbers below show the length of the respective regions. Proposed or known functions for each *cis*-element are described in Table S4

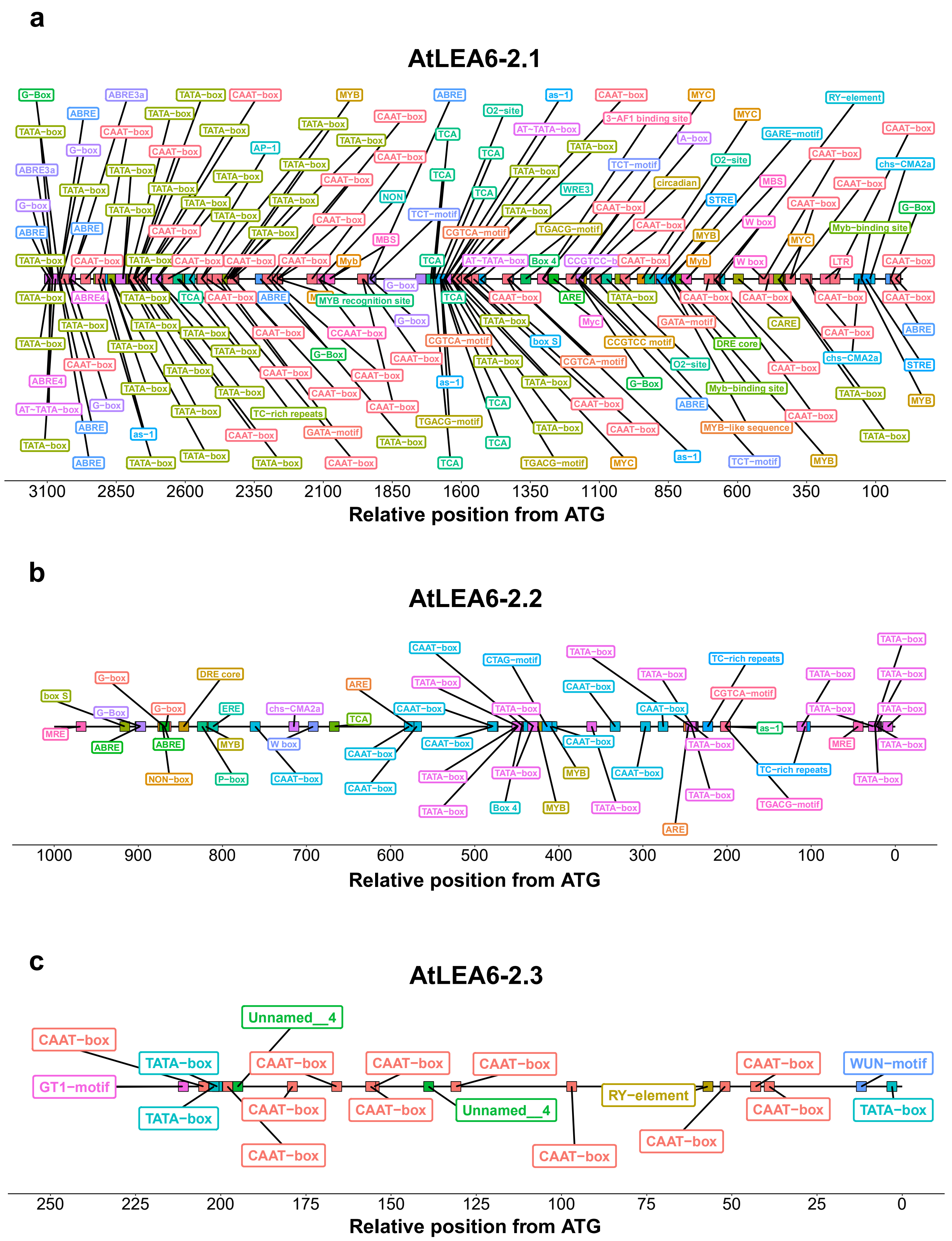

Figure S10. Stress conditions do not affect the germination rate of *atlea6-2.2* mutant. (**a**) Representative scheme of the *AtLEA6-2.2* gene showing the corresponding ORF (grey box), 5' and 3' UTRs (dashed boxes), the oligonucleotides used (arrows), and the T-DNA insertion site. (**b**) PCR analysis to localize the T-DNA insertion and orientation. The letters in the upper part of the figure indicate the oligonucleotide pairs used for each reaction. (**c**) End-point RT-PCR analysis to determine the presence of *AtLEA6-2.2* transcript. *eIF4* transcript was used as loading control. (**d**-**e**) Germination rate of wild-type (Wt) and *atlea6-2.2* mutant (ET9692) under optimal and stress conditions. Germination was quantified by scoring radicle emergence using seeds from homozygous lines plated on MS medium (0.5X) (inset in **d**), or on MS added with mannitol (300 mM) (**d**) or NaCl (200 mM) (**e**). Seeds were stratified for 3 days and incubated in a growth chamber at 22°C. Error bars indicate SD of 4 independent replicates (n = 200), data were fit to a sigmoidal dose-response curve.

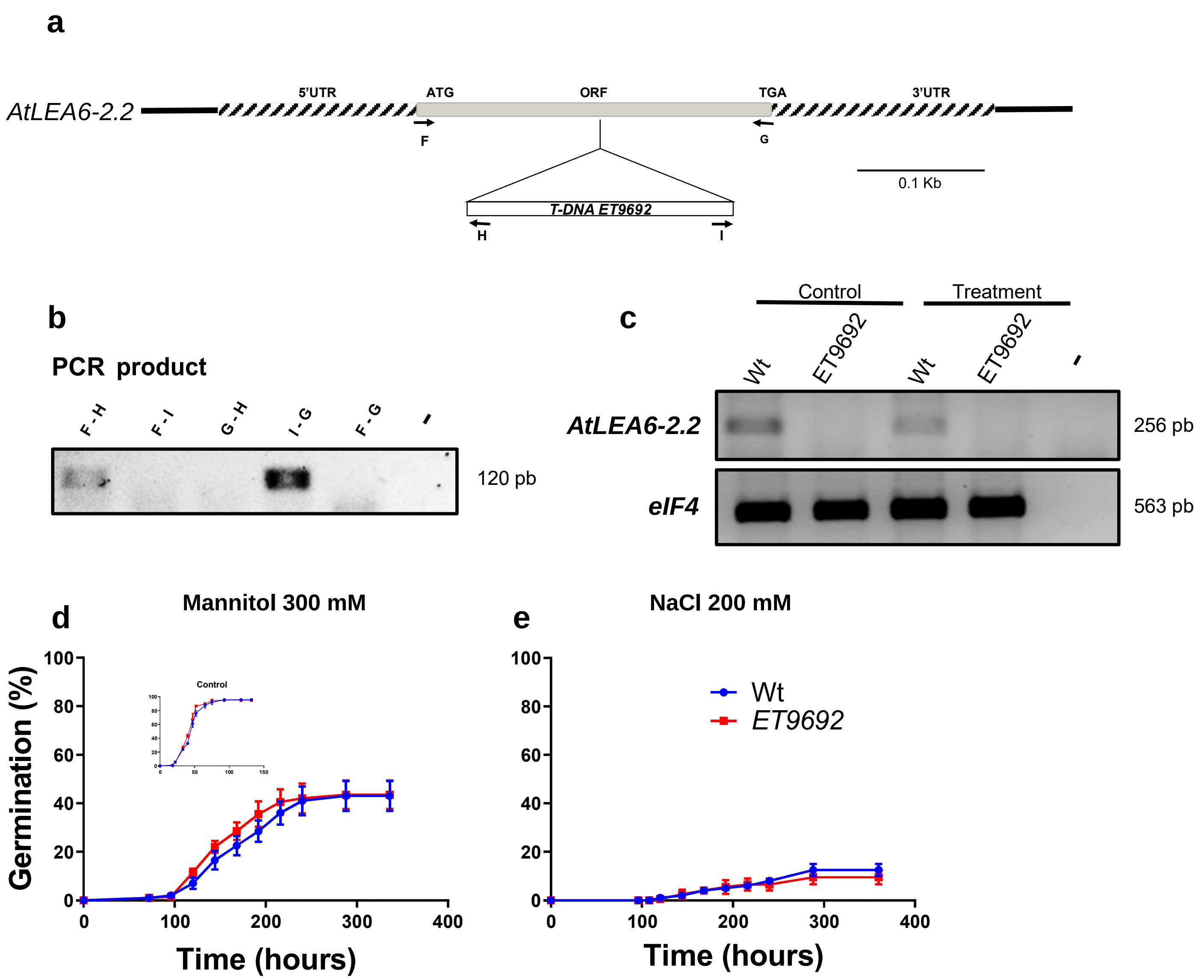

Figure S11. Statistical analysis of the germination rate data presented in Fig. 5. Statistical analysis of germination rates using One-Way ANOVA. Tukey post-hoc test was used to evaluate statistical significance. Raw data used for this analysis is in Supporting data set (germination_data_fig5).

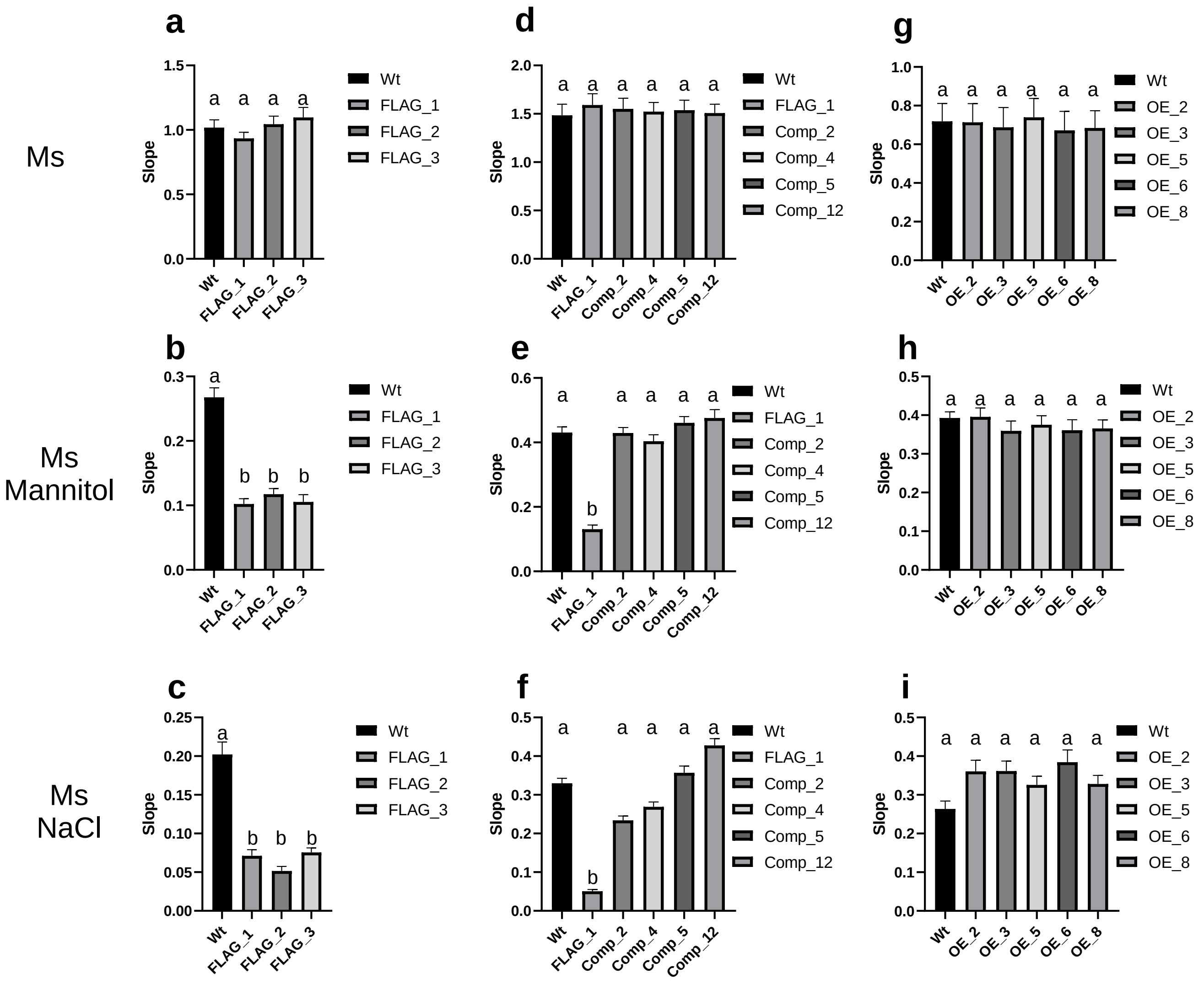

Figure S12. The sensitive phenotype of the *atlea6-2.1* mutant (FLAG_1) under media containing varying concentrations of salt and mannitol exhibits a dose-dependent response. Sensitivity to salt or mannitol increases as the solute concentration in the medium increases. (**a**) Germination under 100 mM mannitol. Inset shows the germination rate of wild-type (Wt) (blue line) and *atlea6-2.1* mutant (FLAG-1) (red line) lines in MS medium (0.5X) without no addition. (**b**) Germination under 200 mM mannitol. (**c**) Germination under 300 mM mannitol. (**d**) Germination under 70 mM mM NaCl. (**e**) Germination under 130 mM NaCl. (**f**) Germination under 200 mM NaCl. Seeds were stratified for 3 d and incubated in a growth chamber at 22°C. Error bars indicate SD of 3 independent replicates (n = 300). Data were fit to a sigmoidal dose-response curve. Germination was quantified by radicle emergence using seeds of homozygous lines. See details in Materials and Methods.

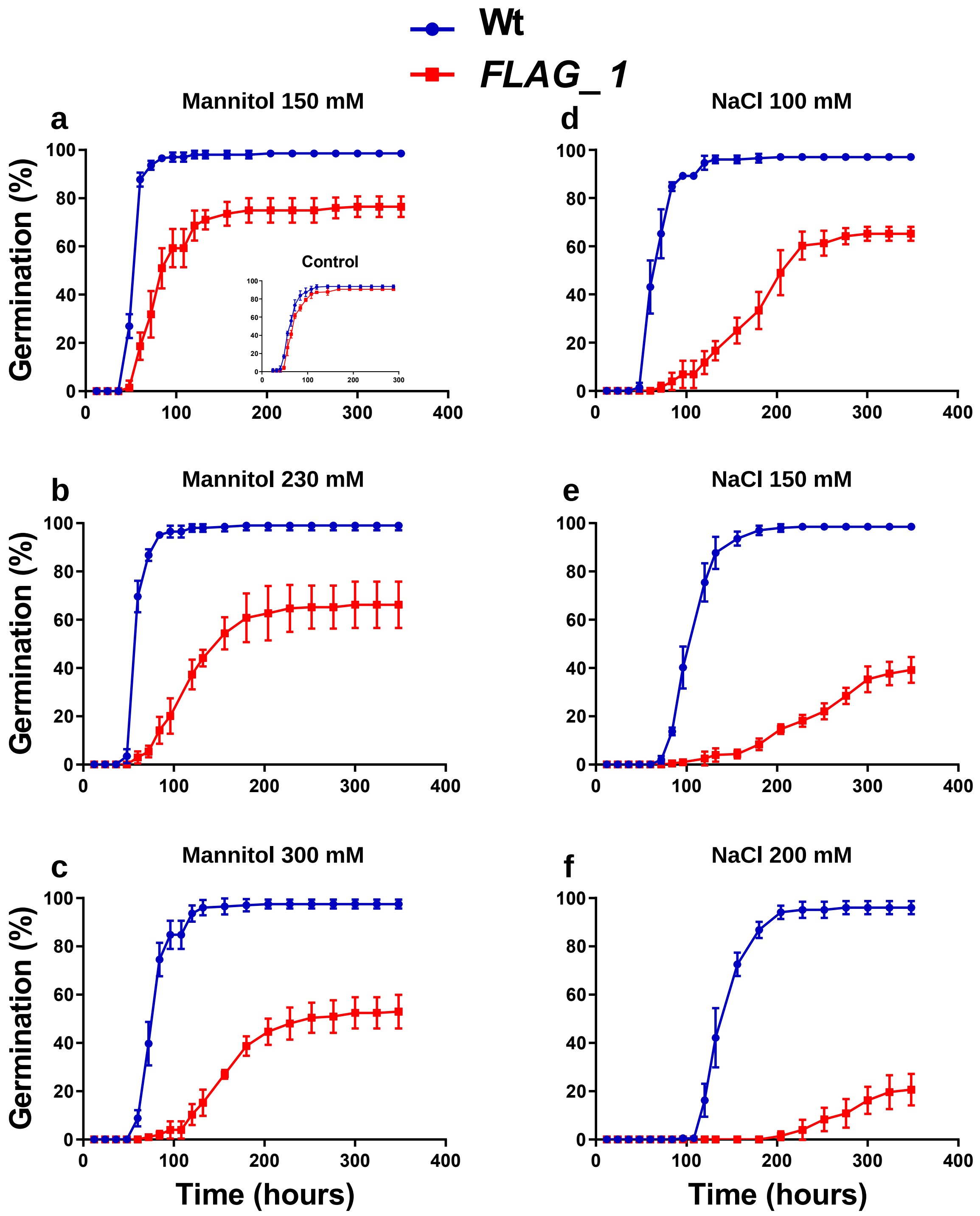

Figure S13. Glass transition onset and endset temperatures are not influenced by the presence or absence of AtLEA6-2.1. (**a**) Glass transition temperature onset values for different seed lines. (**b**) Glass transition temperature endset values for different seed lines. (**c**) Correlation plot of water content versus glass transition temperature midpoint values for all seed lines. (**d**) Correlation plot of water content versus glass former fragility values for all seed lines. Statistics were calculated using a one-way ANOVA and Tukey post-hoc test: *p*-value > 0.05 are statistically not significant, error bars represent standard error (SD) in bar charts and 95% confidence intervals (CI) in correlation plots. ns: non-significant.

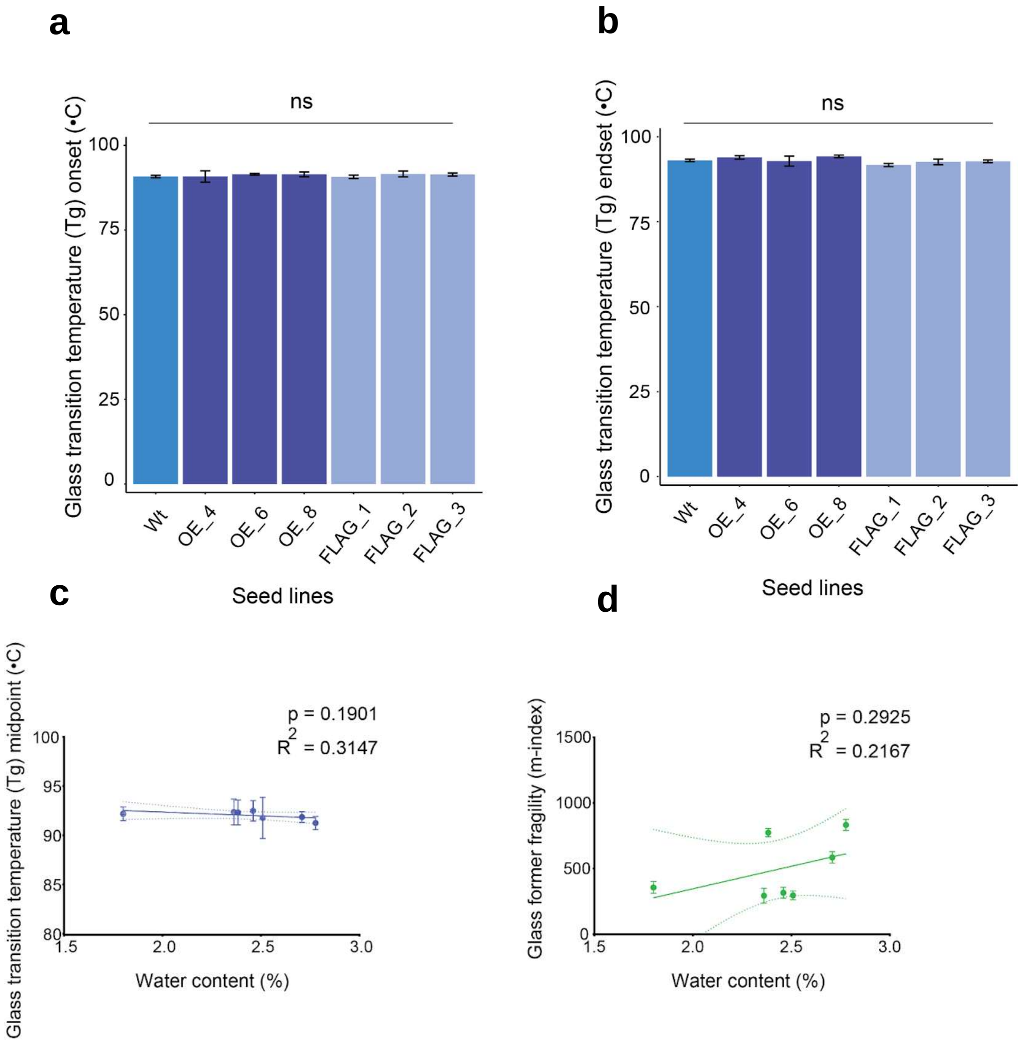

Figure S14. Graphs showing the theoretical isoelectric points (IP) of LEA6 (**a**) and LEA4 (**b**) proteins. IPs were calculated using the CIDER server (Holehouse et al., 2017).

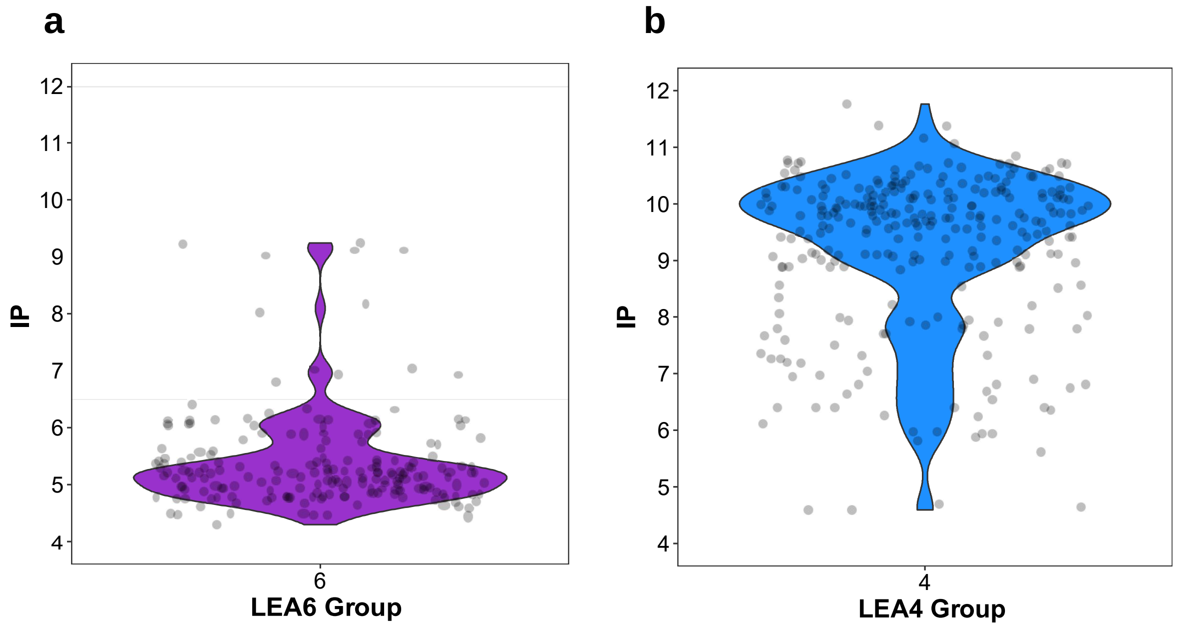

Figure S15. Computed net charge nz of LEA6 and LDHb in an aqueous medium is shown for 3D folded conformations of their polypeptide chains (blue and red, respectively) across different pH values (**a**). Interaction model by docking of the LEA6 and LDHb at pH 7 (**b**) and pH 8 (**c**). Red and blue colors indicate that the electrostatic fields at the molecular surface are negative and positive, respectively. The images on the left show the frontside views, while the right display the backside views. The computed free energy of the LEA6 (blue line) and the LDHb (red line) structures across different pH values (**d**) The free energy and net charge were calculated in APBS (Adaptive Poisson-Boltzmann Solver) software suite (Jurrus et al., 2018).

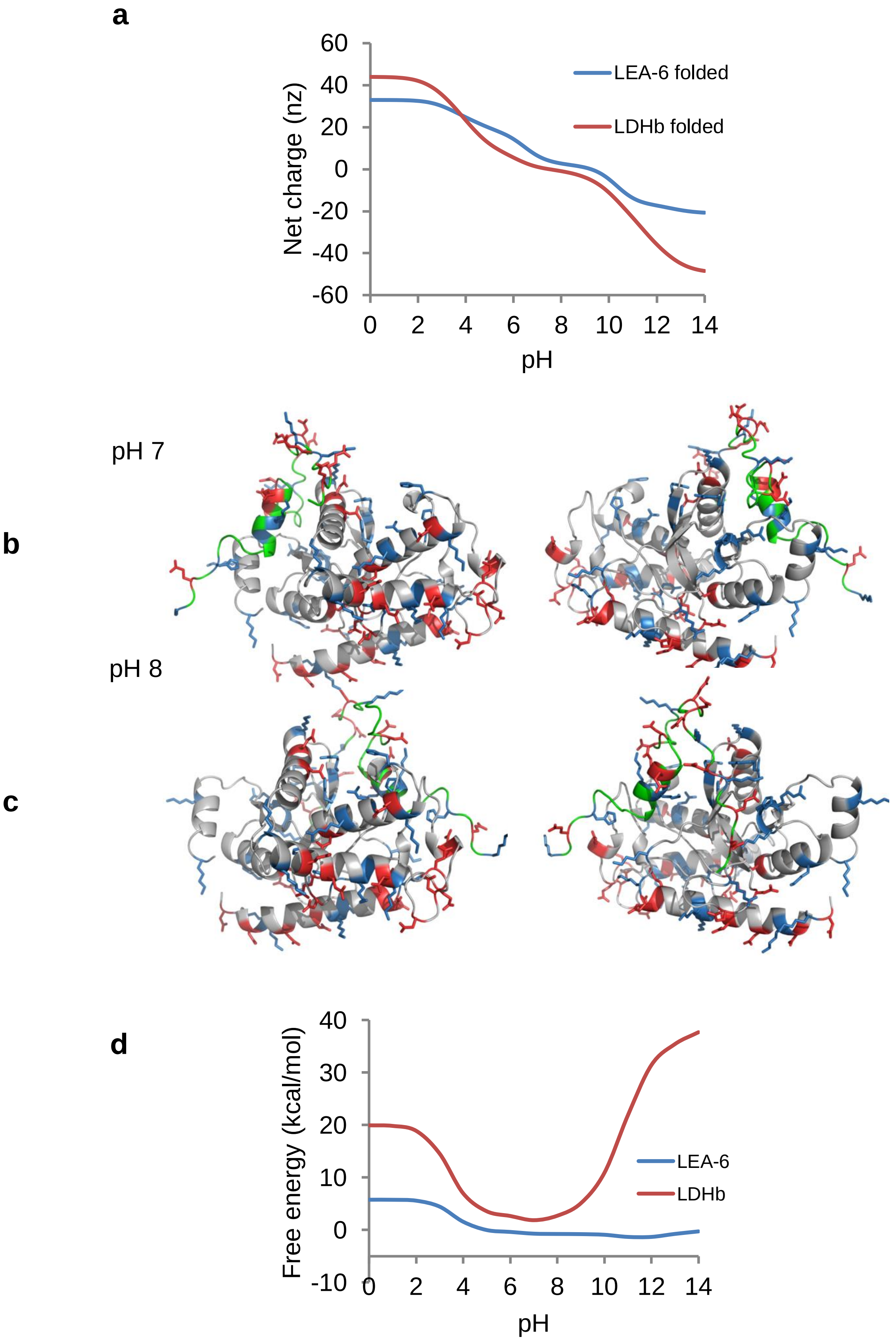

Supplement: Supplementary file 1 — Supplementary Figures Arroyo‐Mosso et al. [file PCE-48-6874-s002.pdf]
