# Supplementary material for: A Group 6 LEA Protein Plays Key Roles in Tolerance to Water Deficit, and in Maintaining the Glassy State and Longevity of Seeds
Source: Plant Cell Environ. 2025 Jun 5;48(9):6874–96. doi: 10.1111/pce.15649 (PMC12319291; doi:10.1111/pce.15649)
Supplement: Supplementary file 3 — supmat. [file PCE-48-6874-s001.docx]

**Supporting Information**

**Figure S1**. Phylogeny of the LEA6 family showing paralogous proteins.

**Figure S2**. Reconciled tree of the LEA6 proteins with the species tree carried out using

Treerecs.

**Figure S3**. Reconciled tree of the LEA6 proteins with the species tree carried out

using SpeciesRax.

**Figure S4**. Phylogeny of the LEA6 family members from the order Brassicales, among the large orders, the one with most species and members.

**Figure S5**. Multiple sequence alignment of 90 sequences of LEA6 proteins highlighting sequence conservation.

**Figure S6**. Multiple sequence alignment of 90 sequences of LEA6 proteins highlighting charged residues.

**Figure S7**. Representative image of Arabidopsis chromosome 2.

**Figure S8.** (**a**) Relative abundance of Arabidopsis LEA6 transcripts across different organs.

**Figure S9.** *cis*-elements in the promoter regions of *AtLEA6* genes.

**Figure S10**. Stress conditions do not affect the germination rate of *atlea6-2.2*

mutant.

**Figure S11**. Statistical analysis of the germination rate data presented in Fig. 5.

**Figure S12**. Dose dependence of the sensitive phenotype of the *atlea6-2.1* mutant (FLAG_1)

**Figure S13**. Glass transition onset and endset temperatures are not influenced by the

presence or absence of AtLEA6-2.1.

**Figure S14**. Graphs showing the theoretical isoelectric points (IP) of LEA6 and LEA4 proteins.

**Figure S15**. Computed net charge nz of LEA6 and LDHb m for their 3D folded conformations across different pH values.

**Table S1**. List of RefSeq IDs of all LEA6 proteins used in this study.

**Table S2**. Support values from RAxML phylogenetic trees.

**Table S3**. List of oligonucleotide sequences used in this study.

**Table S4**. *cis*-elements located in the promoter regions of *AtLEA6* genes.

**Table S5**. Reference names of LEA6 sequences in FASTA format

**Table S6**. Raw germination data of *AtLEA6-2.1* mutants grown under control and stress conditions.

**Table S7**. Raw germination data of FLAG_1 *AtLEA6-2.1* mutant in the dose–response experiments.

**Table S8**. Raw germination data of *atlea6-2.2* under control and stress conditions.

**Table S9**. Raw data corresponding to fresh and dry weight analyses of *AtLEA6-2.1* mutants grown under control and stress conditions.

**Table S10**. Raw root growth data of *AtLEA6-2.1* mutants grown under control and stress conditions.

**Table S11**. Raw data corresponding to aging analysis of *AtLEA6-2.1* mutants.

**Table S12**. Raw data of glassy state property analyses of *AtLEA6-2.1* mutant and wild-type seeds.

**Table S13**. Raw data from *in vitro* dehydration assays.
